# Exosome-shuttled miR-216a-5p from hypoxic preconditioned mesenchymal stem cells repair traumatic spinal cord injury by shifting microglial M1/M2 polarization

**DOI:** 10.1186/s12974-020-1726-7

**Published:** 2020-02-04

**Authors:** Wei Liu, Yuluo Rong, Jiaxing Wang, Zheng Zhou, Xuhui Ge, Chengyue Ji, Dongdong Jiang, Fangyi Gong, Linwei Li, Jian Chen, Shujie Zhao, Fanqi Kong, Changjiang Gu, Jin Fan, Weihua Cai

**Affiliations:** Department of Orthopaedics, The First Affiliated Hospital of Nanjing Medical University, Nanjing, 210029 Jiangsu China

**Keywords:** Spinal cord injury, Exosomes, Hypoxia, Microglia polarization, miR-216a-5p/TLR4 axis

## Abstract

**Background:**

Spinal cord injury (SCI) can lead to severe motor and sensory dysfunction with high disability and mortality. In recent years, mesenchymal stem cell (MSC)-secreted nano-sized exosomes have shown great potential for promoting functional behavioral recovery following SCI. However, MSCs are usually exposed to normoxia in vitro, which differs greatly from the hypoxic micro-environment in vivo. Thus, the main purpose of this study was to determine whether exosomes derived from MSCs under hypoxia (HExos) exhibit greater effects on functional behavioral recovery than those under normoxia (Exos) following SCI in mice and to seek the underlying mechanism.

**Methods:**

Electron microscope, nanoparticle tracking analysis (NTA), and western blot were applied to characterize differences between Exos and HExos group. A SCI model in vivo and a series of in vitro experiments were performed to compare the therapeutic effects between the two groups. Next, a miRNA microarray analysis was performed and a series of rescue experiments were conducted to verify the role of hypoxic exosomal miRNA in SCI. Western blot, luciferase activity, and RNA-ChIP were used to investigate the underlying mechanisms.

**Results:**

Our results indicate that HExos promote functional behavioral recovery by shifting microglial polarization from M1 to M2 phenotype in vivo and in vitro. A miRNA array showed miR-216a-5p to be the most enriched in HExos and potentially involved in HExos-mediated microglial polarization. TLR4 was identified as the target downstream gene of miR-216a-5p and the miR-216a-5p/TLR4 axis was confirmed by a series of gain- and loss-of-function experiments. Finally, we found that TLR4/NF-κB/PI3K/AKT signaling cascades may be involved in the modulation of microglial polarization by hypoxic exosomal miR-216a-5p.

**Conclusion:**

Hypoxia preconditioning represents a promising and effective approach to optimize the therapeutic actions of MSC-derived exosomes and a combination of MSC-derived exosomes and miRNAs may present a minimally invasive method for treating SCI.

## Background

Traumatic spinal cord injury (SCI), with high morbidity and mortality, remains a devastating problem worldwide [1]. It is estimated that the mortality rate of hospitalized acute SCI ranges from 4.4 to 16.7% globally. With the aging population, the number of recorded cases of traumatic SCI caused by falls has increased from 16 to 30.5% since 2012 [2, 3]. Patients often suffer from lasting cognitive or motor dysfunction following SCI that has a devastating effect on their ability to continue with normal life [4]. Primary damage of the spinal cord is related to the destruction of axons and neurons, while secondary damage is caused by neuroinflammation, which can lead to edema, cavitation, and reactive gliosis morphologically [5–8]. At present, long-term treatments mainly deal with symptoms of secondary complications, such as serious neuroinflammation and poor adaptive plasticity following secondary damage [9]. However, because of the blood-brain barrier (BBB), until recently, very few therapeutic drugs or other interventions have been verified to inhibit the development of secondary damage and effectively promote functional recovery after SCI [10].

Following SCI, as one of the main resident cells in the central nervous system (CNS), microglia mediate a cascade of events and play a vital role in the activation and regulation of neuroinflammation [11, 12]. They exert dual effects on neuroinflammation and neurogenesis, depending on their polarization: the classical M1 phenotype secretes pro-inflammatory cytokines including TNF-α, IL-6, and IL-1β, which are harmful to neurogenesis. The alternative M2 phenotype secretes anti-inflammatory cytokines including TGF-β, IL-10, and IL-4, which are favorable to neurogenesis [13, 14]. Thus, efforts should focus on exploring therapeutic strategies to shift microglia from M1 to M2 phenotype as well as suppress detrimental excessive neuroinflammation for the treatment of SCI [15, 16].

Transplantation of mesenchymal stem cells (MSCs) shows therapeutic effects in SCI [17–19]. However, limitations and challenges cannot be ignored when transplanting MSCs directly into target tissues. Studies have reported that the survival rate of transplanted stem cells is very low. Other related risks including immune rejection, cell dedifferentiation, and tumor formation and these limit the underlying application of direct clinical MSC transplantation [20–22]. Recent studies investigating the role of MSCs in tissue regeneration have shown that paracrine mechanisms may be involved in the underlying MSC action mechanism in the treatment of several diseases and exosomes may play an important role in this process [23, 24].

Exosomes are nano-sized liposomes that originate from invagination of endosomal membranes and are important components of the paracrine secretion of cells [25]. They are formed from multivesicular bodies with a diameter of 50–150 nm and are released into the extracellular space through fusion with plasma membranes, while protecting their contents from degradation. They participate in the transport of biochemicals such as proteins, cytokines, mRNAs, and miRNAs and, as a result, play an essential role in intercellular communication through the transfer of genetic material [26–28]. According to ExoCarta, an exosome online database, there are about 8000 proteins known to be related with exosomes. Exosomes have not only specific proteins that depend on cell types but also a subgroup of common and abundant proteins including CD9, CD63, CD81, TSG101, and so on which have been used as positive markers to detect the presence of exosomes regardless of cell types [29, 30]. The specific surface ligands of exosomes ensure that they bind to target cells and deliver their contents, thereby regulating specific biological functions including transmitting intercellular signaling, enhancing angiogenesis, promoting tumor cells proliferation and metastasis, modulating immune responses, and so on [31–33]. Transplantation of exosomes shows similar therapeutic effects and functional properties to directly transplanted stem cells, but there are less significant adverse effects, emphasizing that this treatment strategy could avoid some of the adverse effects seen when transplanting stem cells directly [23, 24, 34]. In recent years, research has focused on the application of MSC-derived exosomes in the field of tissue engineering, inflammation modulation, regeneration medicine etc. [35–38]. Our studies have shown that exosomes derived from MSCs could enhance functional recovery after SCI by inhibiting neuroinflammation and promoting axonal regeneration [39, 40]. A clinical trial was developed to evaluate the effects of MSC-derived exosomes on the possible treatment of type I diabetes mellitus (ClinicalTrials.gov, NCT02138331) and the first patient has successfully been treated with MSC exosomes in graft versus host disease [41].

Oxygen concentration is important in the process of proliferation, differentiation, and self-renewal of MSCs [42, 43]. However, during in vitro culture conditions, MSCs are usually exposed to normoxia (21% O_2_), which differs from the oxygen concentrations found in the body under natural physiological conditions. A large proportion of MSCs exist in a hypoxic environment (≤ 2–8% O_2_) in the body. A recent study isolated exosomes from MSCs that were grown in media similar to that found in peripheral arterial disease (0% FBS, 1% O_2_) and found that the exosomes contained a number of pro-angiogenic factors that may be beneficial to ischemic tissues [43]. A different study, using an infarcted heart model, found that exosomes derived from MSCs after hypoxic treatment, showed increased vascularization, lower apoptosis rates of cardiomyocytes, and increased recruitment of cardiac progenitor cells [44]. Our previous study also showed that ischemic hypoxia preconditioning could suppress cell death in a model of ischemia-reperfusion injury in rats [45]. Indeed, hypoxic preconditioning of MSCs can significantly enhance their biological function and activity, thereby improving the transplantation efficacy of MSCs in the treatment of various disease models [46, 47]. Although some studies have reported that MSC exosomes could suppress the development of inflammation by shifting the microglia/macrophage phenotype in traumatic CNS diseases [48–50], it is still unclear whether MSCs under hypoxic conditions can exert better therapeutic effects to promote SCI functional recovery and whether such enhancement is mediated by exosomal signaling.

Recent studies have focused on exosomal contents including proteins and RNAs and attempted to determine their underlying mechanisms in the treatment of various diseases [26, 51–53]. However, the miRNA in exosomes derived from MSCs under hypoxic conditions and the underlying mechanisms by which these contribute to SCI remains unknown. It has been shown that exosomal miRNAs could exert their regulatory effects on target cells, thus representing a new mode of intracellular communication. Because treatment using hypoxia preconditioning can improve the therapeutic effects of MSCs and modulate specific miRNA expression, we attempted to confirm a role for hypoxic treatment in the enhancement of exosome bioactivity through the regulation of miRNAs [54, 55]. Using a miRNA microarray, miR-216a-5p was found to be the most enriched microRNA in HExos and could shift microglia from the M1 to M2 phenotype in vivo and in vitro. Correspondingly, our results demonstrated that knockdown of miR-216a-5p in HExos (miR^KD^-HExos) could abolish the beneficial effects seen with HExos and overexpression of miR-216a-5p in HExos (miR^OE^-HExos) could enhance the beneficial effects seen with HExos. Meanwhile, we identified Toll-like receptor 4 (TLR4) as the target gene of exosomal miR-216a-5p through online databases, and a series of gain- and loss-of-function analyses were carried out to verify it. In this study, we demonstrated that miR-216a-5p-enriched exosomes, which were released from MSCs under hypoxic preconditioning, could shift microglia from the M1 to M2 phenotype by suppressing the activity of TLR4, thereby regulating the TLR4/NF-κB/PI3K/AKT signaling cascade, and as a result, promote functional recovery following SCI in mice. This finding indicated an underlying mechanism for the application for MSC-derived exosomes under hypoxia and provided a promising therapeutic target for SCI.

## Methods

### Reagents and antibodies

The microglial activator lipopolysaccharide (LPS) was purchased from Sigma-Aldrich (St. Louis, MO, USA). The antibodies use for western blotting in our study included anti-β-actin (Abcam, Cambridge, UK), anti-TSG101 (Abcam), anti-CD9 (Abcam), anti-CD63 (Abcam), anti-CD81 (Cell Signaling Technology, Danvers, MA, USA), anti-iNOS (Abcam), anti-Arg1 (CST), anti-TLR4 (Abcam), anti-p-P65 (CST), anti-MyD88 (CST), anti-p-PI3K (CST), anti-PI3K (CST), anti-p-AKT (CST), and anti-AKT (CST). The antibodies used for immunofluorescence were anti-iNOS (Abcam), anti-Arg1 (CST), anti-Iba1 (Servicebio, Wuhan, China), anti-NF200 (Abcam), and anti-GFAP (Abcam). The secondary antibody was a cyanine 3- or FITC-conjugated secondary antibody (Jackson ImmunoResearch, West Grove, PA). The TNF-α, IL-1β, IL-6, TGF-β, IL-4, and IL-10 ELISA kits were obtained from R&D Systems.

### Cell culture and hypoxia treatment

Bone MSCs (BMSCs) were isolated and cultured as previously reported [39, 56]. BMSCs from passages 3–5 were used for further experiments. They were cultured in a normoxic cell incubator at 37 °C, 5% CO_2_, and 21% O_2_ or in a hypoxic cell incubator set at 1% O_2_ in exosome-depleted fetal bovine serum (FBS)-containing (System Biosciences, Mountain View, CA, USA) media for 48 h.

For identification of BMSCs, Alizarin Red, Oil Red O, and Alcian Blue stains were used to identify osteogenic, adipogenic, and chondrogenic differentiation, respectively. The BMSCs at passages 3–5 were cultured in OriCell™ osteogenic, adipogenic, or chondrogenic differentiation media, respectively (Cyagen, Guangzhou, China). For identification of BMSC markers, flow cytometry was carried out using fluorescein isothiocyanate (FITC)-conjugated or phycoerythrin (PE)-conjugated antibodies (human anti CD34, anti-CD45, anti-CD73, anti-CD105) (BD Biosciences Pharmingen, San Jose, CA). PE-IgG1 and FITC-IgG1 isotypic immunoglobulins were used as isotype controls. Fluorescence signals were sorted using a flow cytometer (FACSCalibur, BD Biosciences, USA) and the results were analyzed using FlowJo software.

Primary microglia were obtained as previously reported [57, 58]. Briefly, brains were removed from newborn mice (1–3 days old) and carefully cut into 0.5–1-mm^3^ pieces. The cut pieces were then added to 0.25% trypsin-EDTA solution and incubated for 10 min with gentle shaking. Following termination of the trypsinization reaction, the digested tissues were centrifuged at 300×*g* for 5 min and the tissue pellets were resuspended in DMEM/F12. Following filtration with a 100-μm nylon mesh, the final single-cell suspension was cultured in T75 flasks precoated with poly-l-lysine (Sigma) to obtain the primary mixed glial cell cultures. Microglia reach maturity after 14 days of culture in vitro. The mature microglia were removed by shaking the flasks at 200 rpm for 2 h at room temperature. The microglial supernatants were collected and cultured in 6- or 24-well culture plates precoated with poly-l-lysine and cultured at 37 °C, 5% CO_2_-humidified atmosphere. The medium was changed every 3 days. The primary microglia were stimulated with LPS (1 μg/ml) for 24 h to induce a pro-inflammatory phenotype. Exosomes (200 μg/ml) from different groups were then added and co-cultured with the primary microglia.

The BV2 microglial cell line was purchased from the Cell Bank of the Chinese Academy of Science (Shanghai, China). Cell lines were cultured in DMEM/high glucose media containing 10% FBS and 1% pen/strep. LPS (1 μg/ml) was co-cultured with BV2 microglia for 24 h followed by the addition of exosomes (200 μg/ml) in the medium in different groups.

### Exosome isolation and identification

When BMSCs reached 80% confluency, the culture medium was replaced with exosome-depleted FBS for an additional 48 h and cultured under normoxic or hypoxic conditions. The medium was collected and centrifuged at 300×*g* for 10 min, then 2000×*g* for 10 min at 4 °C. Following centrifugation, a 0.22-μm sterile filter (Steritop™ Millipore, Burlington, MA) was used to filter the cell supernatant from the whole cells and cellular debris. The filtered supernatant was then applied to the upper compartment of an Amicon Ultra-15 Centrifuge Filter Unit (Millipore) and centrifuged at 4000×*g* until the volume was reduced to ~ 200 μL in the upper compartment. The ultra-filtered supernatant was then washed twice with PBS and re-filtered to another 200 μL. To purify the exosomes, the liquid was loaded onto the top of a 30% sucrose/D_2_O cushion in a sterile Ultra-Clear™ tube (Beckman Coulter, Asphalt, CA, USA) and centrifuged at 100,000×*g* for 60 min at 4 °C in an optima L-100 XP Ultracentrifuge (Beckman Coulter). The fraction containing the BMSC-Exos (under normoxic conditions) was recovered using an 18-G needle, then diluted in PBS, and centrifuged at 4000×*g* at 4 °C in a centrifugal filter unit until the final volume reached 200 μL. Exosomes were either stored at − 80 °C or used immediately for downstream experiments.

A Nanosight LM10 System (Nanosight Ltd., Navato, CA) was used to analyze the distribution of vesicle diameters from the Exos and HExos. The morphology of the acquired exosomes under normoxia and hypoxia was observed using a transmission electron microscope (TEM; Tecnai 12; Philips, Best, The Netherlands). Western blotting was used to determine specific exosome surface markers such as TSG101, CD9, CD63, and CD81.

BMSC-Exo protein concentration was determined using a bicinchoninic acid protein assay (BCA; Thermo Fisher Scientific, Waltham, MA). Absorbance was read at 562 nm with a microplate reader (ELx800; Bio-Tek Instruments, Inc., Winooski, VT).

### Exosome uptake by BV2 microglia

Fluorescent labeling of Exos and HExos was carried out according to the manufacturer’s instructions. Briefly, 4 mg/mL Dil solution (Molecular Probes, Eugene, OR, USA) was added to PBS containing exosomes and incubated. Excessive dye from labeled exosomes was removed by ultracentrifugation at 100,000×*g* for 1 h at 4 °C. Exosome pellets were then washed three times by re-suspending the pellet in PBS with a final wash and resuspension in PBS. These Dil-labeled exosomes were co-cultured with BV2 microglia for 24 h, and the cells were then washed with PBS and fixed in 4% paraformaldehyde. The uptake of Dil-labeled Exos and HExos by BV2 microglia was then observed by laser confocal microscopy and the fluorescence intensity of Dil was measured with ZEN lite software at different time points within the two groups.

### Vector constructs, lentivirus production, and cell transfections

LV2-mmu-miR-216a-5p-mimic vector (miR^OE^) and the LV2-mmu-miR-216a-5p-inhibitor vector (miR^KD^) were constructed by lentiviral vectors (GenePharma, Shanghai, China). We also constructed a negative control with the LV2 empty lentiviral (miR-NC^OE^ and miR-NC^KD^). BMSCs, grown to 40–50% confluence, were infected by using lentiviral vectors at an appropriate multiplicity of infection (MOI). Vectors for the overexpression and shRNA targeting of mouse TLR4 using lentiviral gene transfer were constructed by GenePharma (Shanghai, China). The scrambled lentiviral construct was used as a negative control. BV2 microglia and primary microglia were transfected with the lentiviral vectors (Vector, TLR4, shNC, and shTLR4). Lipofectamine 3000 reagent (Invitrogen) was used for transfection according to the manufacturer’s instructions.

### In vitro detection of miR-216a-5p transfer

BMSCs were transfected with 5′-carboxyfluorescein (FAM)-labeled miR-216a-5p mimics, miR-216a-5p inhibitor and their corresponding negative controls (GenePharma, Shanghai, China) with Lipofectamine 3000. After that, exosomes were extracted from the culture medium in the four different groups and added into target BV2 cells. After co-incubation, BV2 cells were fixed with 4% PFA and permeabilized with 0.05% Trition X-100, and stained with DAPI (Thermo Fisher Scientific). Images were acquired using a confocal microscope to observe the green signaling intensity in the target BV2 cells.

### Quantitative real-time PCR

TRIzol® reagent (Invitrogen, Carlsbad, CA, USA) was used to extract total RNA from cells and exosomes. Complementary DNA (cDNA) was synthesized using a reverse transcription system (Toyobo, Osaka, Japan) and qRT-PCR was carried out with SYBR Green PCR master mix (Applied Biosystems, Foster City, CA) on an ABI 7900 fast real-time PCR system (Applied Biosystems, Carlsbad, USA). Expression levels were normalized to the internal controls (β-actin or U6) and the relative expression levels were evaluated using the 2^−ΔΔCT^ method. The specific primers for miR-216a-5p, miR-99b-5p, miR-301a, miR-126, miR-210-3p, U6, TLR4, iNOS, TNF-α, IL-1β, Arg1, CD206, YM1/2, and β-actin were purchased from RiboBio Co, Ltd. (Guangzhou, China). The primer sequences are listed in Additional file 7: Table S1.

### Exosomal miRNA microarray assay

The microRNA arrays for Exos and HExos were carried out by OE Biotech Company (Shanghai, China). Three samples were processed for each exosome. The fragmentation mixtures were hybridized to an Agilent-Mouse microRNA array 21.0 (8*60 K, Design ID:070155). For microarray analysis, the Affymetrix (Santa Clara, CA, USA) miRNA 4.0 platform was used. The sample labeling, microarray hybridization, and washing were performed based on the manufacturer’s instructions (Agilent Technologies Inc., Santa Clara, California, USA). Differentially expressed miRNAs were identified using a fold change cut-off value of ≥ 1.5 set for both up- and downregulated genes.

### Western blot analysis

Proteins were extracted from cells and treated with RIPA lysis and extraction buffer (KeyGen Biotechnology, Nanjing, China). Protein concentration was determined using the BCA method. Equal amounts of protein were separated by SDS-PAGE, transferred to PVDF membranes (EMD Millipore Corp., Burlington, MA), and incubated overnight at 4 °C with primary antibodies followed by blocking with bovine serum albumin (BSA, 5%, v/v). Membranes were then incubated for 120 min at room temperature with the secondary antibody. Reacting bands were visualized using ECL reagent (Thermo Fisher Scientific), and the density of protein bands was semi-quantified using ImageJ (National Institutes of Health, Bethesda, MD, USA).

### Luciferase reporter assay

Sequences corresponding to the 3′-UTR of TLR4 mRNA and containing the wild-type (WT) or mutated (MUT) miR-216a-5p binding sequence were synthesized by GeneScript (Nanjing, China). We cloned these sequences into the FseI and XbaI restriction sites of the pGL3 luciferase control reporter vector (Promega, USA) to generate the TLR4 3′-UTR reporter constructs (pGL3-WT-TLR4 and pGL3-MUT-TLR4). BV2 microglia and primary microglia were seeded in 24-well plates and incubated for 24 h before transfection. BV2 microglia and primary microglia transfected with miR^OE^ or negative control were seeded into 96-well plates and co-transfected with 100 ng of pGL3-WT-TLR4 or pGL3-MUT-TLR4 3′-UTR. Firefly and Renilla luciferase signals were determined using a Dual-Luciferase® Assay Kit (Promega, Madison, WI, USA).

### Isolation of RISC-associated RNA

BV2 microglia overexpression miR-216a-5p or miR-NC^OE^ were fixed with 1% formaldehyde, followed by chromatin fragmentation, lysed in NETN buffer and then incubated with Dynabeads Protein A (Invitrogen) supplemented with clone 2A8 antibody (Millipore), anti-Pan-Ago, or IgG control for immunoprecipitation. The immunoprecipitated RNA was released by proteinase K digestion and extracted by phenol/chloroform/isopropyl alcohol. RNA was isolated by glycogen ethanol precipitation and treated with DNase I.

### ELISA

To evaluate the expression levels of pro-inflammatory cytokines including TNF-α, IL-1β, and IL-6 and anti-inflammatory cytokines including TGF-β, IL-4, and IL-10 in the injured spinal cord, the tissues were isolated at 3 days after SCI. Liquid nitrogen was added to the homogenizer to smash the injured spinal cord. We then added lysis buffer, which included 1 mM EDTA, 1% Triton X-100, 1 mM phenylmethylsulphonyl fluoride, 150 mM NaCl, 10 mM Tris pH 8.0, and 5 μl/ml protease inhibitor into the lysates and incubated for 1 h at 4 °C. The lysates were centrifuged at 3000 rpm for 30 min, and the supernatants collected to measure the cytokine concentration using ELISA kits, according to the manufacturers’ protocols.

In BV2 and primary microglial culture medium, the pro- and anti-inflammatory cytokines were measured using ELISA kits according to the manufacturers’ protocols. Optical density or fluorescence was measured with a plate reader.

### Preparation of contusive spinal cord injury mouse model and experimental groups

The animal protocols were approved by the Animal Committee of the First Affiliated Hospital of Nanjing Medical University. An SCI model of male mice (C57BL/6, 6–8 weeks old) was established, as previously described. After anesthetizing the animal, a laminectomy was used to expose the spinal cord at T10, and a spinal cord impactor (68,097, RWD, CA, USA) was used to create injury by dropping a rod (weighing 5 g) onto the spinal cord from a height of 6.5 cm. Muscles were sutured immediately after administration, and the skin was closed. Bladders of animals were manually voided three times per day until reflexive control of bladder function was restored.

Mice were randomly assigned into several groups (*n* = 8/group for each time point). Mice were subjected to SCI, followed by tail vein injection of Exos, HExos, miR-NC^OE^-HExos, miR^OE^-HExos, miR-NC^KD^-HExos, miR^KD^-HExos (200 μg of total protein of exosomes precipitated in 200 μL PBS), or an equal volume of PBS (200 μL) immediately following SCI.

### Functional locomotor scores

Neurological function was quantified at 1, 3, 7, 14, and 28 days post-injury using the Basso Mouse Scale (BMS) for locomotion. Scoring ranged from 0 (complete paraplegia) to 9 (normal function). Footprint analysis was also performed as previously described. The forelimbs and hindlimbs of the mice were dipped in blue and red dyes, respectively. The stride lengths and widths were measured and analyzed only when the mice ran at a constant velocity. Each mouse was assessed by two independent examiners blinded to the treatment regimen.

### Electrophysiology

To evaluate functional recovery after SCI, motor-evoked potentials (MEPs) of mice 4 weeks post-injury were analyzed using electromyography according to previous studies [59–61]. Mice were firstly anesthetized with 10% chloral hydrate solution. After that, a stimulation electrode was applied to the rostral ends of the surgically exposed spinal cord, the recording electrode was placed in the biceps femoris flexor cruris, the reference electrode was inserted at the distal tendon of the muscle in the hindlimb, and the ground electrode was placed subcutaneously. A single square wave stimulus (0.5 mA, 0.5 ms, 1 Hz) was applied. Peak-to-peak amplitude was used to detect the nerve conduction function in the hindlimb of mice.

### Magnetic resonance imaging

Three animals in each group were randomly selected for magnetic resonance imaging (MRI) examination at Day 3 post-injury. Mice were anesthetized with halothane (3–4% induction, 1.5–2% maintenance) in oxygen (0.4 L/min) and nitrogen (0.6 L/min). Anesthetized mice were placed on a fixation system in a prone position. Experiments were performed on a small animal MRI system (Bruker BioSpec 7 T/20 USR; Bruker AXS GmbH, Karlsruhe, Germany). The sequence protocol was carried out with the following parameters: T_2_-weighted; 256 × 256 matrix; slice thickness, 1 mm; intersection gap, 1 mm; echo time/repetition time: 27/3000 ms; rapid acquisition with relaxation enhancement factor, 16; and flip angle, 90°. T_2_-weighted images were acquired in the sagittal and axial planes by ParaVision (version 6.0.1, Bruker BioSpec; Bruker AXS GmbH).

### Immunofluorescence staining

The mouse hearts were perfused with 0.9% saline followed by 4% paraformaldehyde. The spinal segments surrounding the lesion center were removed and fixed overnight in 4% paraformaldehyde. Following dehydration in 15% and 30% sucrose solutions, the samples were frozen and cut into 10 mm thick sections for subsequent experiments. For tissue immunofluorescence staining, the frozen spinal sections were blocked with 10% BSA and incubated overnight at 4 °C with the following primary antibodies: anti-NF200, anti-GFAP, anti-Iba1, anti-iNOS, and anti-Arg1, followed by secondary antibodies for 1 h at room temperature. For cell immunofluorescence staining, the cells were fixed in 4% paraformaldehyde for 30 min, then permeabilized with 0.05% Triton X-100, and finally blocked with 5% BSA. Primary antibodies (anti-Iba1, anti-iNOS, and anti-Arg1) were added and the cells were incubated overnight at 4 °C. This was followed by incubation with the following secondary antibodies. After triple washing with PBS, nuclei were stained with DAPI (Thermo Fisher Scientific) and fluorescent images were acquired using a fluorescence microscope (AxioVertA1 and ImagerA2). Four different areas of gray matter near the traumatic lesion were selected as the near-injury area and four different areas at least 10-mm distance from the traumatic lesion were chosen as the far-injury area. The average intensity of NF200 for each area was measured by ZEN lite software. Data are expressed as the percentage of intensity increase or decrease in the near-injury area compared with the far-injury area.

### Statistical analyses

All experiments were performed in at least three independent biological replicates. Data are shown as mean ± standard deviation. GraphPad software 7.0 and SPSS 19.0 were used for statistical analysis. We used the Student’s *t* test for two-group comparisons and one-way or two-way ANOVA for more than two-group comparison to calculate the *P* values. A value of *P* < 0.05 was considered statistically significant.

## Results

### Identification of bone mesenchymal stem cells

BMSCs were isolated from mice as described above. In passage 3, BMSCs were identified by morphology and flow cytometry. Cells adopted a spindle-like shape at 80–90% confluency (Additional file 1: Figure S1A). Alizarin Red, Oli Red O and Alcian Blue staining were applied to identify the osteogenic, adipogenic and chondrogenic differentiation of BMSCs, respectively (Additional file 1: Figure S1B). Flow cytometry analysis was used to confirm that BMSCs were positive for CD73 and CD105 but negative for CD34 and CD45 (Additional file 1: Figure S1C).

### Hypoxia promotes exosome release from bone mesenchymal stem cells

**Fig. 1:**
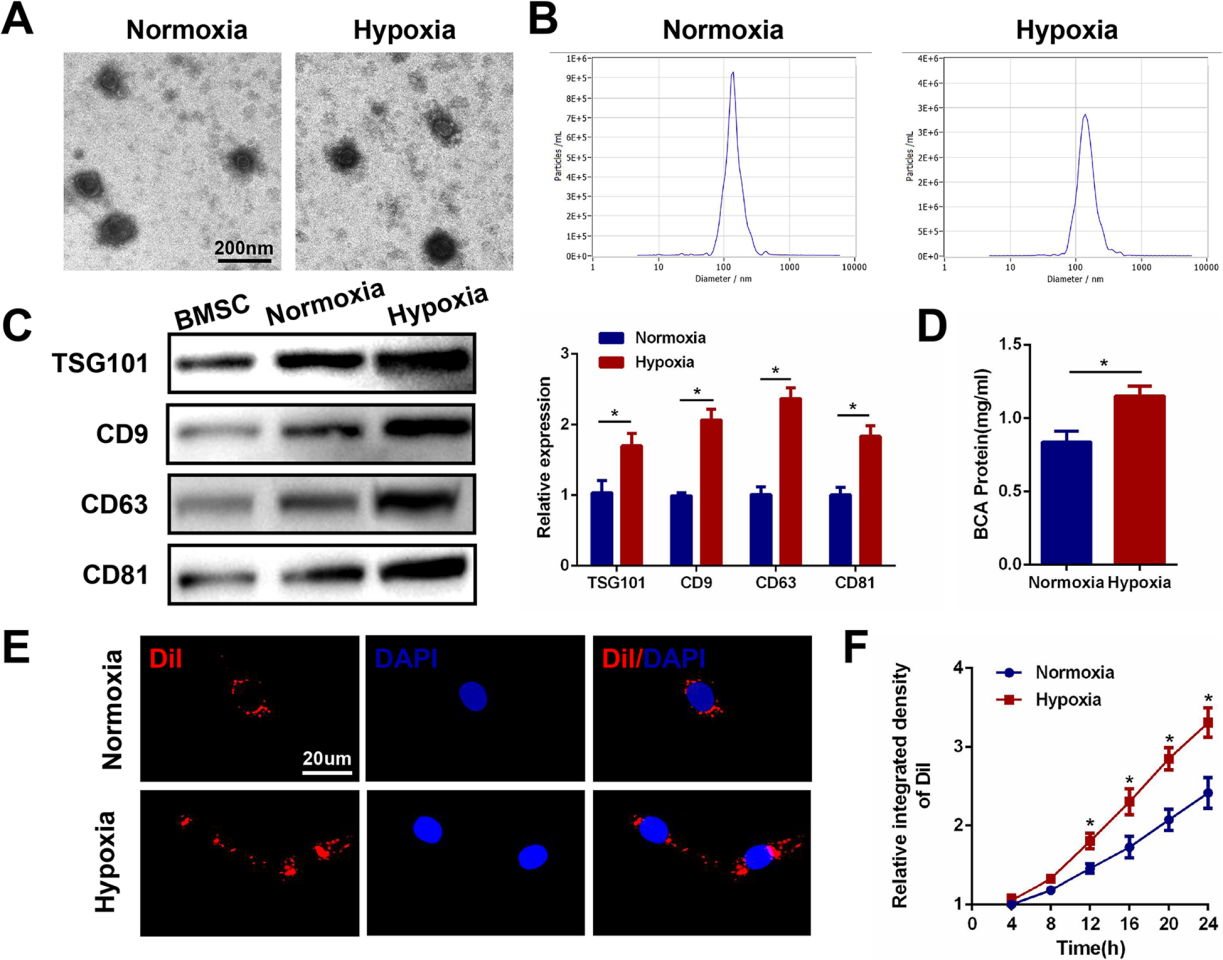
Hypoxia promotes exosome release from MSCs. **a** Morphology of Exos and HExos under TEM. **b** NTA analysis of Exos and HExos revealed that exosomes from the two groups exhibit similar size ranges (50–150 nm). **c** Western blot analysis of exosomal proteins including TSG101, CD9, CD63 and CD81. **d** Exosome protein concentration in the two groups using the BCA assay. **e** Uptake of the red fluorescence dye Dil-labeled Exos and HExos into BV2 microglia. **f** Statistical evaluation of fluorescence intensities in the two groups. ^*^P < 0.05

The content and function of exosomes is dependent upon the cell of origin, suggesting that intercellular communication through exosomes is a dynamic system, and can be adapted depending upon the conditions of the producing cell. Changes in oxygen concentration affect many of the distinctive characteristics of stem and progenitor cells and can deliver biological information by internalization in neighboring or distant cells. On this basis, we determined whether the hypoxic condition of MSCs could influence the exosomes that they release. BMSCs were seeded under normoxic and hypoxic (1% O_2_) conditions, respectively and isolated from serum-free media after 48 h of incubation. They were then analyzed using an electron microscope, nanoparticle tracking analysis (NTA) and western blot. TEM revealed typically rounded nanoparticles ranging in size from 50 to 150 nm in diameter and NTA showed a similar size distribution (average 121.6 nm versus 125.3 nm) in both the normoxia and hypoxia groups (Fig. 1a, b). No morphological difference was observed between the two groups with regard to their size, shape, or electron density. Western blot revealed the presence of exosome surface markers including TSG101, CD9, CD63, and CD81. Increased protein levels of TSG101, CD9, CD63, and CD81 were observed in exosomes after exposure to 1% O_2_ for 48 h (Fig. 1c). Moreover, the protein concentration of exosomes derived from hypoxic BMSCs was significantly higher when compared with those from the normoxic controls (Fig. 1d). Hypoxic conditioning induced a significantly increased release of exosomes compared with the normoxic controls.

### The differential uptake of exosomes by BV2 microglia is dependent on oxygen status

To examine whether exosomes derived from normoxic or hypoxic conditions were taken up differentially by BV2 microglia, a Dil dye was used to label the exosomes and then co-cultured with target BV2 for 24 h. Fluorescence microscopy was used to monitor the rate of exosome uptake by BV2 in real time. As shown in Fig. 1e, the number of exosomes taken up by BV2 was significantly greater in the hypoxia group compared with the normoxic control group. Figure 1 f demonstrates a clear statistically significant difference between the two groups after 12 h, suggesting that exosomes derived under hypoxic conditions are more easily taken up by microglia.

### HExos administration promoted functional behavioral recovery after SCI compare with Exos

**Fig. 2:**
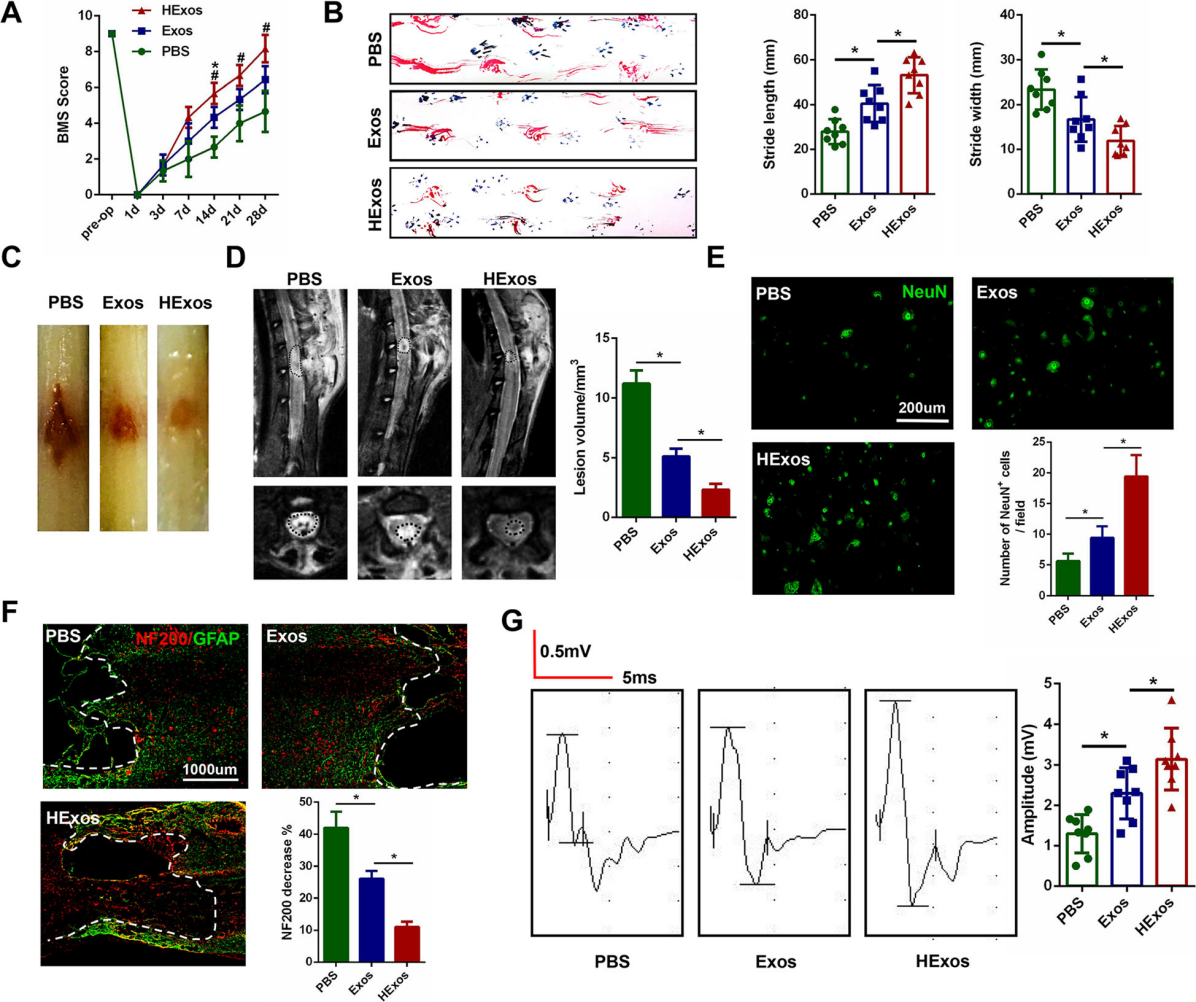
HExos administration promoted functional behavioral recovery following SCI in vivo*.***a** BMS was used to functionally grade the mice in the PBS, Exos and HExos groups up to 28 days post-injury (*n* = 8/group). **b** Representative footprints of an animal walking 28 days after SCI and quantification of the footprints analysis findings in each mouse. Blue: frontpaw print; red: hindpaw print (*n* = 8/group). **c** Gross morphology of spinal cord (*n* = 8/group). **d** Representative sagittal and coronal MRI images (*n* = 3/group). **e** Representative immunostaining images of the NeuN-positive cells in different groups after SCI and the number of NeuN-positive cells were calculated (*n* = 8/group). **f** Representative immunostaining images of NF200 (red) and GFAP (green) in the injured lesion areas of spinal cord at day 28 post-injury. The dashed lines indicate the lesion boundaries (*n* = 8/group). **g** MEP analysis was performed as an electrophysiological assessment in different groups at day 28 post-injury (*n* = 8/group). ^*^P < 0.05 between the PBS and Exos groups, ^#^*P* < 0.05 between the Exos and HExos groups

Our previous studies have demonstrated that exosomes derived from MSCs can promote functional recovery post-injury. In this study, to investigate if exosomes derived from hypoxic preconditioned MSCs could exert more beneficial effects on motor function after SCI compared with those under normoxic conditions, we first assessed the functional recovery of mice treated with PBS, Exos or HExos using the BMS. As indicated in Fig. 2a, mice in the Exos group showed better functional improvement compared with mice in the PBS group, which was consistent with previous studies. However, in the present study, we noticed that there was a significant increase in BMS in the HExos group compared with the Exos group. The co-ordination assessments of forepaw-hindpaw movements in the three groups also verified the BMS results. Mice that were treated with Exos showed significantly faster gait recovery and improved motor co-ordination compared with the PBS group mice, and this favorable effect was increased in the HExos group (Fig. 2b). The traumatic lesion site was clearly visible in the gross morphology of the injured spinal cords (Fig. 2c). Following treatment with either Exos or HExos, the lesion area was notably smaller than that in the PBS group. These results also indicated that the lesion area in the HExos group was significantly smaller than that in the Exos group. Randomly selected mice from each group at day 3 after SCI were analyzed by MRI. Representative images are shown in Fig. 2d and MRI results confirmed that injection with HExos remarkably reduced the lesion area compared with the Exos group. To further investigate the effects of HExos on neurons, we stained the neuronal marker NeuN around the injured spinal cord lesion to observe the number and morphology of neurons in the three groups. As indicated in Fig. 2e, NeuN-positive neurons were increased in quantity in the Exos group compared with the PBS group. Moreover, the number of neurons in the HExos group was significantly greater than that in the Exos group. We further examined the density or status of axons in the injured spinal cord to elucidate the anatomical basis of the observed locomotor recovery. By immunostaining analysis of the 200-kDa subunit of a neurofilament (NF200), which is a well-known neuronal marker, we found that the decrease in the staining against NF200 in the lesion areas compared with the distant area, as assessed by average pixel intensity values, was much lower in the HExos group than in the Exos group at day 28 post-injury (Fig. 2f). To further study motor functional behavioral recovery, electrophysiological analyses were applied. As shown in Fig. 2g, MEP amplitudes were higher in the HExos group than in the Exos group at day 28 post-injury, indicating that hindlimbs exhibited better recovery of electrophysiological functions with administration of HExos. Taken together, these results indicate that transplantation of both Exos and HExos could promote functional behavioral recovery following SCI in mice and that these beneficial effects were much more evident following HExos treatment.

### HExos administration promoted microglia/macrophage polarization from M1 to M2 phenotype in vivo

**Fig. 3:**
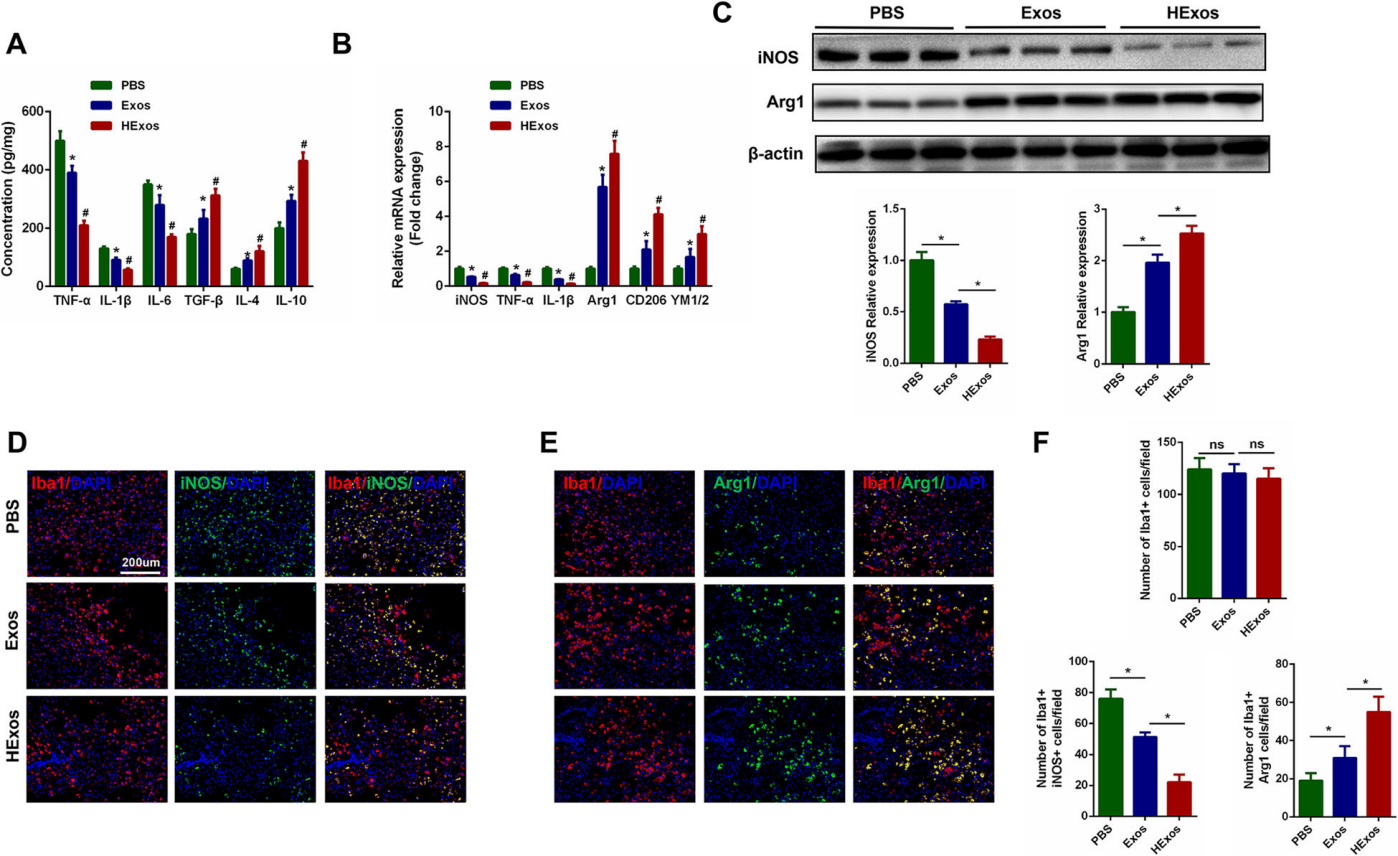
Administration of HExos following SCI promoted microglia/macrophage polarization from M1 to M2 phenotype in vivo. **a** The concentration of pro-inflammatory and anti-inflammatory cytokines in PBS, Exos and HExos groups (*n* = 8/group). **b** The mRNA expression levels of M1- and M2-related genes were detected by qRT-PCR (*n* = 8/group). **c** The protein levels of M1- and M2-related genes were detected by western blot analysis (*n* = 8/group). **d**–**f** Representative immunostaining image of Iba1 (red) and iNOS/Arg1 (green) in the injured spinal cord lesion areas at day 3 post-injury and the analysis of iNOS/Arg1-positive microglia/macrophage in the traumatic lesion area (*n* = 8/group). ^*^P < 0.05 between the PBS and Exos groups, ^#^*P* < 0.05 between the Exos and HExos groups

Three days after SCI, we measured, by ELISA, the concentration of pro-inflammatory cytokines TNF-α, IL-1β, and IL-6 and anti-inflammatory cytokines TGF-β, IL-4, and IL-10 in the spinal cord tissues in the different groups. The results showed that administration of both Exos and HExos could significantly decrease the concentrations of the pro-inflammatory cytokines and elevate the concentrations of the anti-inflammatory cytokines compared with the control PBS group. However, treatment with HExos could greatly promote the secretion of anti-inflammatory cytokines and inhibit the secretion of pro-inflammatory cytokines when compared with Exos alone (Fig. 3a). As microglia/macrophage can have two different phenotypes, we therefore queried whether administration of HExos could polarize microglia/macrophage from M1 towards the M2 phenotype after SCI. The gene expression of M1 (iNOS, TNF-α, IL-1β) and M2 genes (Arg1, CD206, YM1/2) was analyzed by qRT-PCR. As shown in Fig. 3b, the M2 gene expression in the Exos and HExos groups was significantly increased, and M1 gene expression decreased compared with the PBS group. Meanwhile, M2 gene expression was higher and M1 expression lower in the HExos group compared with the Exos group. Western blot analysis also confirmed the qRT-PCR results (Fig. 3c). Furthermore, we evaluated the characteristic polarization of microglia/macrophage after SCI in different groups using the representative M1-assocaited iNOS and M2-associated Arg1 markers for double immunofluorescent staining together with Iba1, which detects microglia/macrophage in the injured spinal cord. As shown in Fig. 3d–f, there were no significant differences in the number of Iba1-positive microglia/macrophage among the three groups, but a marked decrease in the iNOS-positive microglia and a higher level of Arg1 in the microglia/macrophage was observed in the lesion areas at day 3 post-injury in the Exos and HExos groups compared with the PBS group. Interestingly, the number of iNOS-positive microglia/macrophage tended to be lower and Arg1-positive microglia/macrophage higher in the HExos group compared with the Exos group, emphasizing the effect of HExos treatment in the M1/2 polarization of microglia/macrophage in vivo. Consequently, these results demonstrated that HExos had a significant effect on the ratio of anti-inflammatory to pro-inflammatory phenotype after SCI and could shift microglial/macrophage polarization from M1 to M2 phenotype.

### HExos shifted the microglia from M1 to M2 phenotype in BV2 microglia and primary microglia in vitro

**Fig. 4:**
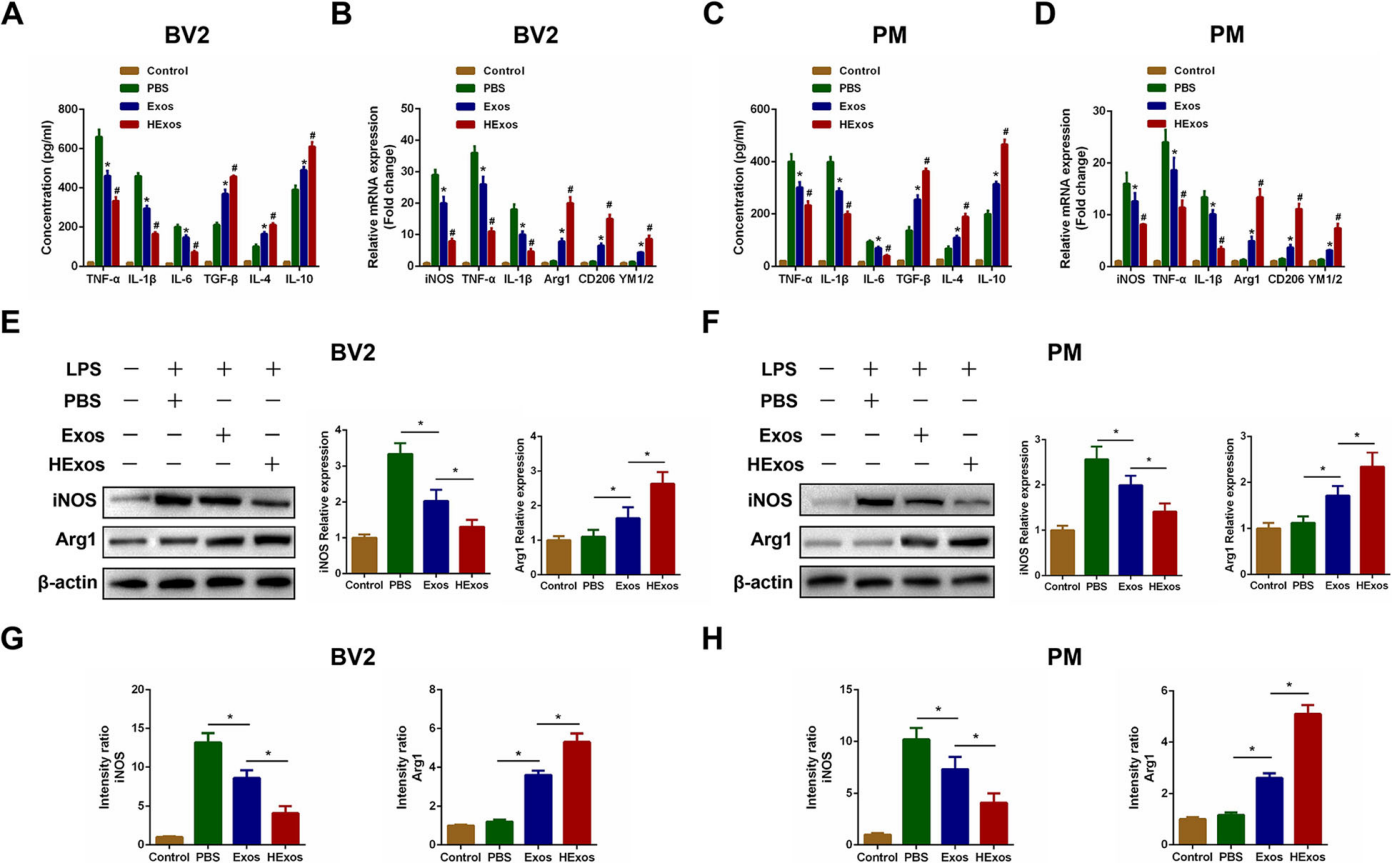
HExos shifted the microglia from M1 to M2 phenotype in BV2 and primary microglia in vitro. **a** The concentrations of pro-inflammatory and anti-inflammatory cytokines in BV2 microglia in the Control, PBS, Exos and HExos groups. **b** The mRNA expression levels of M1- and M2-related genes were detected by qRT-PCR in BV2 microglia in the Control, PBS, Exos and HExos groups. **c** The concentrations of pro-inflammatory and anti-inflammatory cytokines in primary microglia. **d** The mRNA expression levels of M1- and M2-related genes were detected by qRT-PCR in primary microglia. **e** and **f** The protein expression levels of M1- and M2-related genes were detected by western blot in BV2 and primary microglia in the different groups. **g** and **h** Quantification of the immunofluorescence intensity of iNOS and Arg1 from five different fields in each group. ^*^P < 0.05 between the PBS and Exos groups, ^#^*P* < 0.05 between the Exos and HExos groups

To determine whether HExos exert similar therapeutic effects to those observed in vivo, LPS was added to culture systems for 24 h prior to the addition of PBS, Exos, or HExos in order to induce an inflammatory micro-environment to mimic the situation of SCI in vivo. About 48 h later, we detected the concentration of pro-inflammatory cytokines TNF-α, IL-1β, and IL-6 and anti-inflammatory cytokines TGF-β, IL-4, and IL-10 in the culture supernatants in BV2 microglia and primary microglia. We found that both Exos and HExos inhibited the concentration of pro-inflammatory cytokines and promoted the secretion of anti-inflammatory cytokines in vitro (Fig. 4a, c). Moreover, HExos exerted more beneficial effects compared with treatment with Exos. To further investigate whether HExos could directly regulate microglial polarization in vitro, we detected the expression levels of M1-related genes (iNOS, TNF-α, IL-1β) and M2-related genes (Arg1, CD206, YM1/2) in the different groups. We found that administration of both Exos and HExos could decrease the expression of M1 markers and increase the expression of M2 markers and that HExos are more powerful in shifting microglia to M2 polarization compared with Exos alone, which was similar to the results in vivo (Fig. 4b, d). Analysis by western blot confirmed these results (Fig. 4e, f). Using immunofluorescence (Fig. 4g, h, Additional file 2: Figure S2), we showed that administration of HExos significantly affected the expression levels of iNOS and Arg1 in BV2 microglia and primary microglia. These combined results suggest that HExos plays a robust role in shifting microglial polarization from M1 to M2 in BV2 and primary microglia in vitro, which confirmed the results observed in vivo.

### MiR-216a-5p is upregulated in HExos and transferred to BV2 microglia and primary microglia by exosomes

**Fig. 5:**
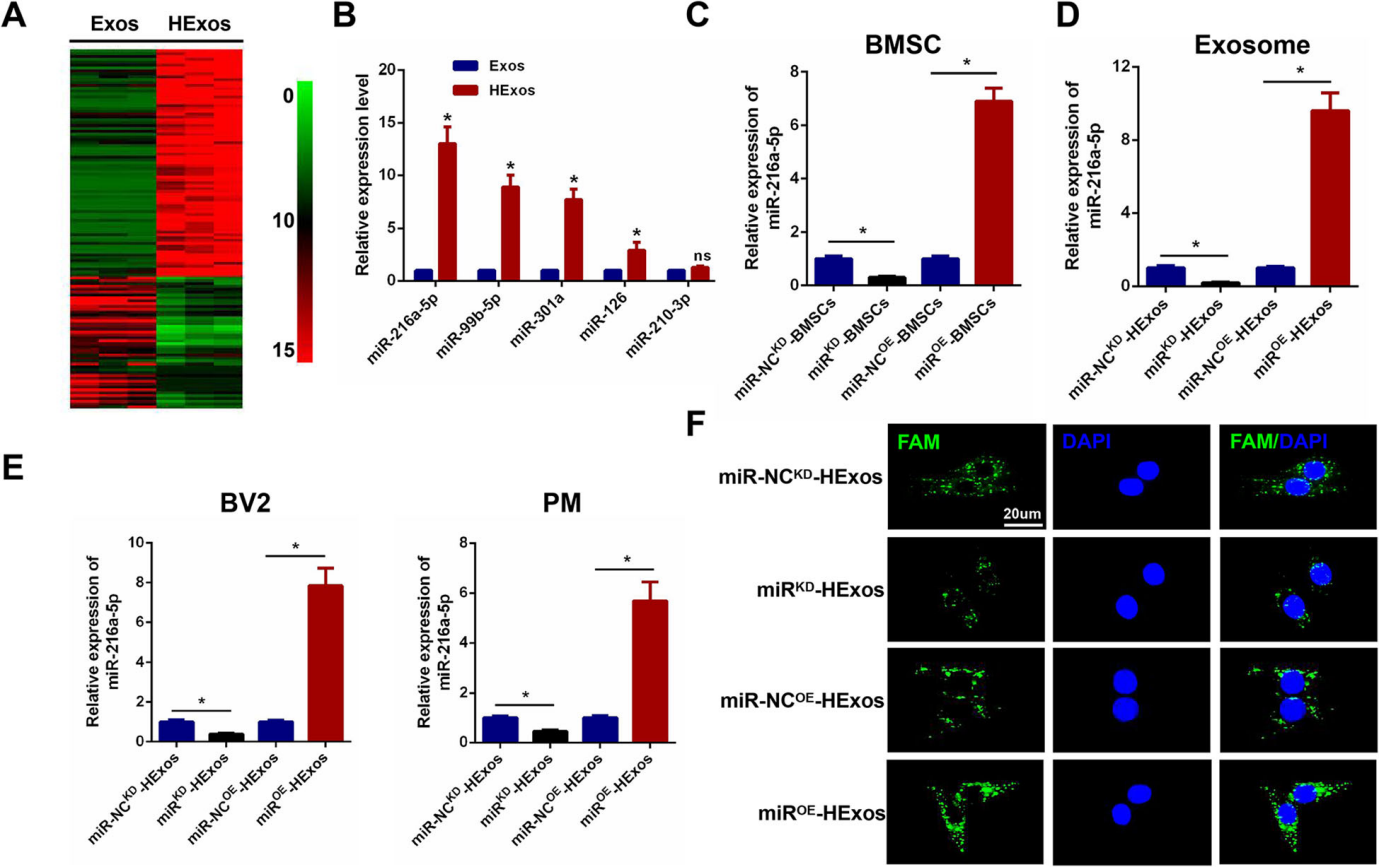
MiR-216a-5p is upregulated in HExos and transferred to BV2 and primary microglia by exosomes. **a** Heat map of the 80 upregulated and 46 downregulated miRNAs with a ≥ 1.5-fold difference between Exos and HExos derived from BMSCs. **b** Comparison of the top five elevated miRNAs including miR-216a-5p, miR-99b-5p, miR-301a, miR-126 and miR-210-3p between Exos and HExos using qRT-PCR. **c** miR-216a-5p overexpression and knockdown in BMSCs and the efficiency confirmed using qRT-PCR. **d** The relative expression level of miR-216a-5p in exosomes derived from hypoxic BMCSs transfected with miR^OE^, miR-NC^OE^, miR^KD^ and miR-NC^KD^. **e** Expression level of miR-216a-5p in target BV2 microglia and primary microglia after administering miR-NC^KD^-HExos, miR^KD^-HExos, miR-NC^OE^-HExos and miR^OE^-HExos. **f** Representative images of FAM-labeled exosomal miR-216a-5p internalized by BV2 microglia after administration of miR-NC^KD^-HExos, miR^KD^-HExos, miR-NC^OE^-HExos and miR^OE^-HExos. ^*^P < 0.05

Both in vitro and in vivo analyses revealed that HExos promoted functional recovery and shifted microglial polarization from M1 to M2 phenotype when compared with Exos alone. A number of previous studies have shown that miRNAs are one of the main functional components of exosomes and may play a crucial role in cell communication and regulation of biological function. Based on the above results, we isolated RNA from Exos and HExos derived from BMSCs, carried out microarray profiling of the miRNAs derived from the exosomes and compared them between the two groups. The miRNA microarray analysis (Fig. 5a) showed that 80 miRNAs were upregulated and 46 downregulated in the HExos group compared with the Exos group (≥ 1.5-fold, *P* < 0.05). Based on these miRNA profiling data, we went on to select the top five upregulated miRNAs including miR-216a-5p, miR-99b-5p, miR-301a, miR-126, and miR-210-3p and validated their expression further using qRT-PCR in vitro. Four miRNAs including miR-216a-5p, miR-99b-5p, miR-301a, and miR-126 from the five selected were significantly upregulated in HExos compared with Exos (Fig. 5b). Based on our microarray and in vitro qRT-PCR results, we concentrated on miR-216a-5p, which showed the most significantly increased expression in HExos and determined whether HExos shifted microglia from M1 to M2 phenotype by the transfer of miR-216a-5p. To gain a mechanistic insight into the role of exosomal miR-216a-5p in HExos-induced shift of microglia phenotype in SCI, we constructed miR-216a-5p overexpression (miR^OE^) and knockdown (miR^KD^) BMSCs using a lentiviral-based method as well as the corresponding negative control (miR-NC^OE^ and miR-NC^KD^). The transfection efficiency was confirmed using qRT-PCR (Fig. 5c). Exosomes were isolated from miR-NC^KD^-BMSCs, miR^KD^-BMSCs, miR-NC^OE^-BMSCs, and miR^OE^-BMSCs named miR-NC^KD^-HExos, miR^KD^-HExos, miR-NC^OE^-HExos, and miR^OE^-HExos, respectively. A significant decrease in the expression of miR-216a-5p in miR^KD^-HExos compared with the miR-NC^KD^-HExos and an evident increase in the expression of miR-216a-5p in miR^OE^-HExos compared with the miR-NC^OE^-HExos was observed (Fig. 5d). Furthermore, the miR-216a-5p expression level in the target BV2 and primary microglia in the miR^KD^-HExos treatment group showed a dramatic decrease in expression compared with the miR-NC^KD^-HExos treatment group. The miR-216a-5p expression levels in the target BV2 and primary microglia in the miR^OE^-HExos treatment group showed an increase in expression compared with the miR-NC^OE^-HExos treatment group (Fig. 5e). Similar to our results using qRT-PCR, the FAM-labeled miR-216a-5p, which was contained in exosomes, was internalized into BV2 microglia, as visualized by confocal microscopy (Fig. 5f). The immunofluorescence data also demonstrated that after treatment with miR^KD^-HExos, FAM-labeled miR-216a-5p immunofluorescence intensity was significantly lower than that of miR-NC^KD^-HExos in BV2 microglia. Meanwhile, administration of miR^OE^-HExos led to a significantly increased immunofluorescence compared with miR-NC^OE^-HExos in BV2 microglia. Taken together, these data indicate that hypoxic MSC-derived exosomal miR-216a-5p can be transferred to target BV2 microglia and primary microglia.

### HExos shifted microglia/macrophage polarization from M1 to M2 phenotype through delivering miR-216a-5p in vivo

**Fig. 6:**
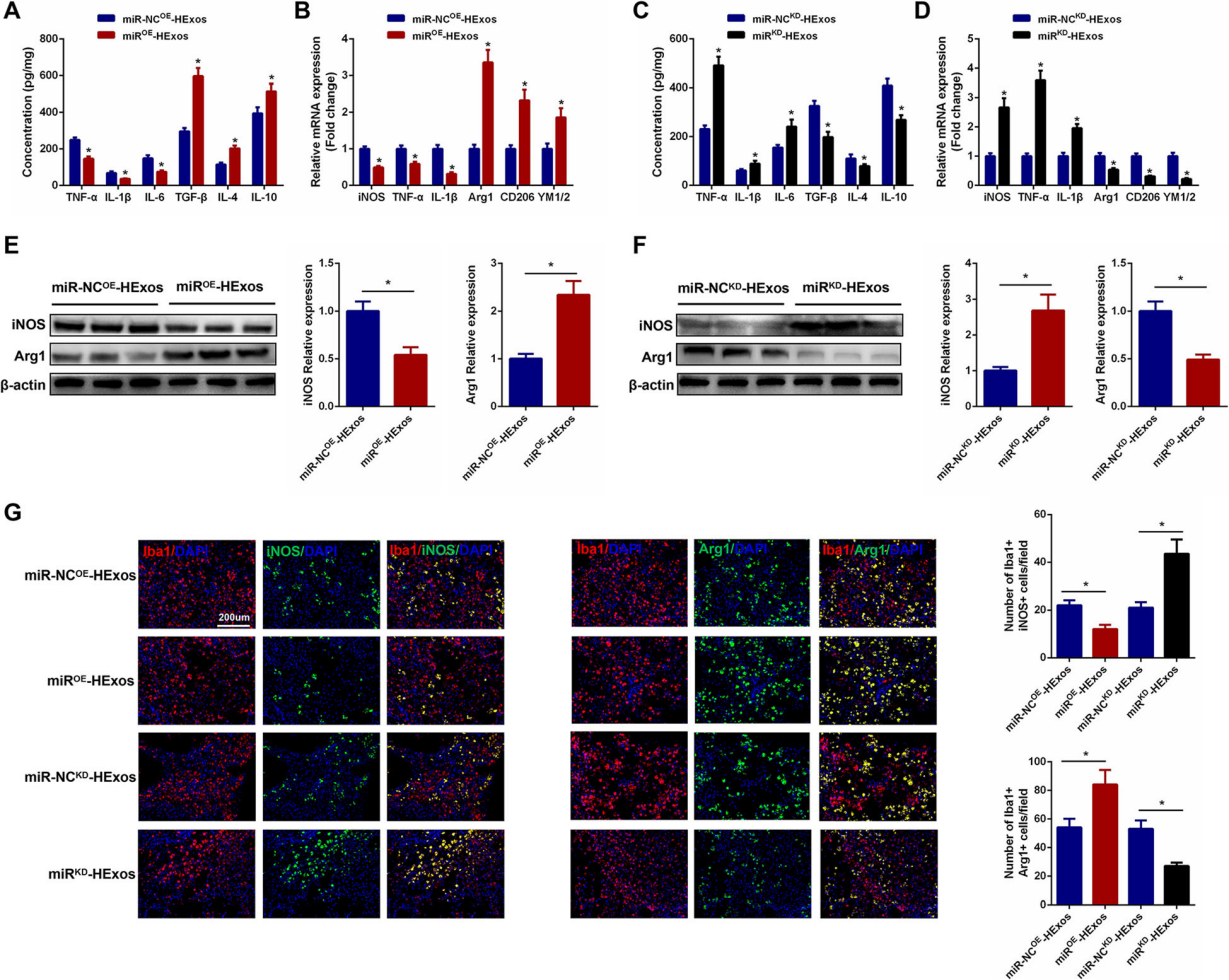
HExos shifted microglia/macrophage polarization from M1 to M2 phenotype by delivering miR-216a-5p in vivo. **a** and **c** The concentrations of pro-inflammatory and anti-inflammatory cytokines in miR-NC^OE^-HExos, miR^OE^-HExos, miR-NC^KD^-HExos and miR^KD^-HExos groups (*n* = 8/group). **b** and **d** The mRNA expression levels of M1- and M2-related genes were detected by qRT-PCR (*n* = 8/group). **e** and **f** The protein levels of M1- and M2-related genes were detected by western blot analysis (*n* = 8/group). **g** Representative immunostaining image of Iba1 (red) and iNOS/Arg1 (green) in the injured spinal cord lesion areas at day 3 post-injury and the analysis of iNOS/Arg1-positive microglia/macrophage in the traumatic lesion area (*n* = 8/group). ^*^P < 0.05

To study the role of miR-216a-5p in the development of HExos-mediated functional behavioral recovery as well as microglial/macrophage polarization after SCI in vivo, we carried out several experiments. Firstly, through a series of functional behavioral experiments including BMS score, footprints analysis, and lesion volume analysis, we found that administration of miR^OE^-HExos could promote functional recovery and reduce lesion volume. In contrast, treatment of miR^KD^-HExos could abolish the beneficial functional effects seen with HExos (Additional file 3: Figure S3). These above results indicated that HExos promoted functional behavioral recovery through delivering miR-216a-5p. Then, we continued to investigate that functional role of miR-216a-5p in the mediation of microglia/macrophage polarization. As shown in Fig. 6 a and c, the concentration of pro-inflammatory cytokines was downregulated and anti-inflammatory cytokines were upregulated in the spinal cord tissues when administrating miR^OE^-HExos compared with miR-NC^OE^-HExos. However, the results were the opposite with miR^KD^-HExos treatment. To detect the effects of miR-216a-5p on microglial/macrophage polarization, M1 and M2 marker levels were detected by qRT-PCR and western blot analysis. The results showed that miR^OE^-HExos facilitated the polarization of microglia/macrophage from M1 to M2 following SCI and miR^KD^-HExos accounted for the opposite effects (Fig. 6b, d, e, f). Accordingly, the immunofluorescence results confirmed the results obtained by qRT-PCR and western blot analysis (Fig. 6g).

### HExos shifted polarization from M1 to M2 in BV2 microglia and primary microglia through shuttling miR-216a-5p in vitro

**Fig. 7:**
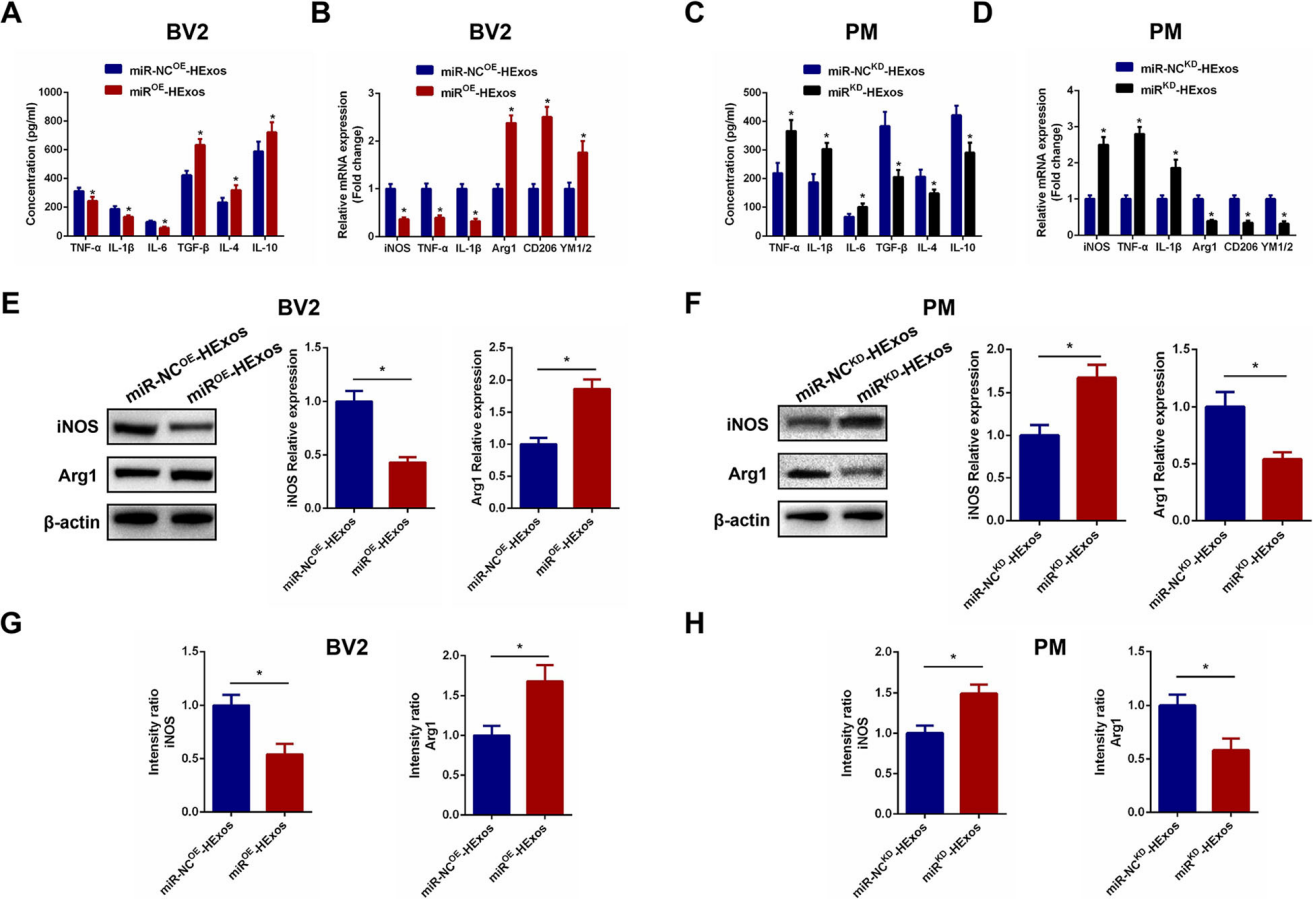
HExos shifted polarization from M1 to M2 in BV2 and primary microglia by shuttling miR-216a-5p in vitro. **a** The concentrations of pro-inflammatory and anti-inflammatory cytokines in BV2 microglia in the miR-NC^OE^-HExos and miR^OE^-HExos groups. **b** The mRNA expression levels of the M1- and M2-related genes were detected by qRT-PCR in BV2 microglia in the miR-NC^OE^-HExos and miR^OE^-HExos groups. **c** The concentrations of pro-inflammatory and anti-inflammatory cytokines in primary microglia in the miR-NC^KD^-HExos and miR^KD^-HExos groups. **d** The mRNA expression levels of M1- and M2-related genes were detected by qRT-PCR in primary microglia in the miR-NC^KD^-HExos and miR^KD^-HExos groups. **e** and **f** The protein expression levels of M1- and M2-related genes were detected by western blot in BV2 and primary microglia in the different groups. **g** and **h** Quantification of the immunofluorescence intensity of iNOS and Arg1 from five different fields in each group. ^*^P < 0.05

To explore the underlying mechanism for HExos shuttling of miR-216a-5p to modulate the microglia phenotype, we carried out a series of experiments in the BV2 and primary microglia in vitro. In these experiments, we administered miR-NC^OE^-HExos and miR^OE^-HExos into BV2 microglia and miR-NC^KD^-HExos and miR^KD^-HExos into primary microglia. Using ELISAs, we found that the pro-inflammatory cytokines decreased and anti-inflammatory cytokines increased in the miR^OE^-HExos group (Fig. 7a). The results were the opposite for administration of miR^KD^-HExos (Fig. 7c). qRT-PCR (Fig. 7b, d), western blot analysis (Fig. 7e, f), and immunofluorescence (Fig. 7g, h, Additional file 4: Figure S4) confirmed that miR^OE^-HExos administration had the ability to shift the M1 phenotype to M2 in BV2 microglia and miR^KD^-HExos treatment had the opposite effect in primary microglia. Taken together, these results demonstrated that HExos shifted the M1 phenotype to M2 in BV2 microglia and primary microglia by shuttling miR-216a-5p in vitro.

### Exosomal miR-216a-5p regulates TLR4 by directly targeting the 3′-UTR

**Fig. 8:**
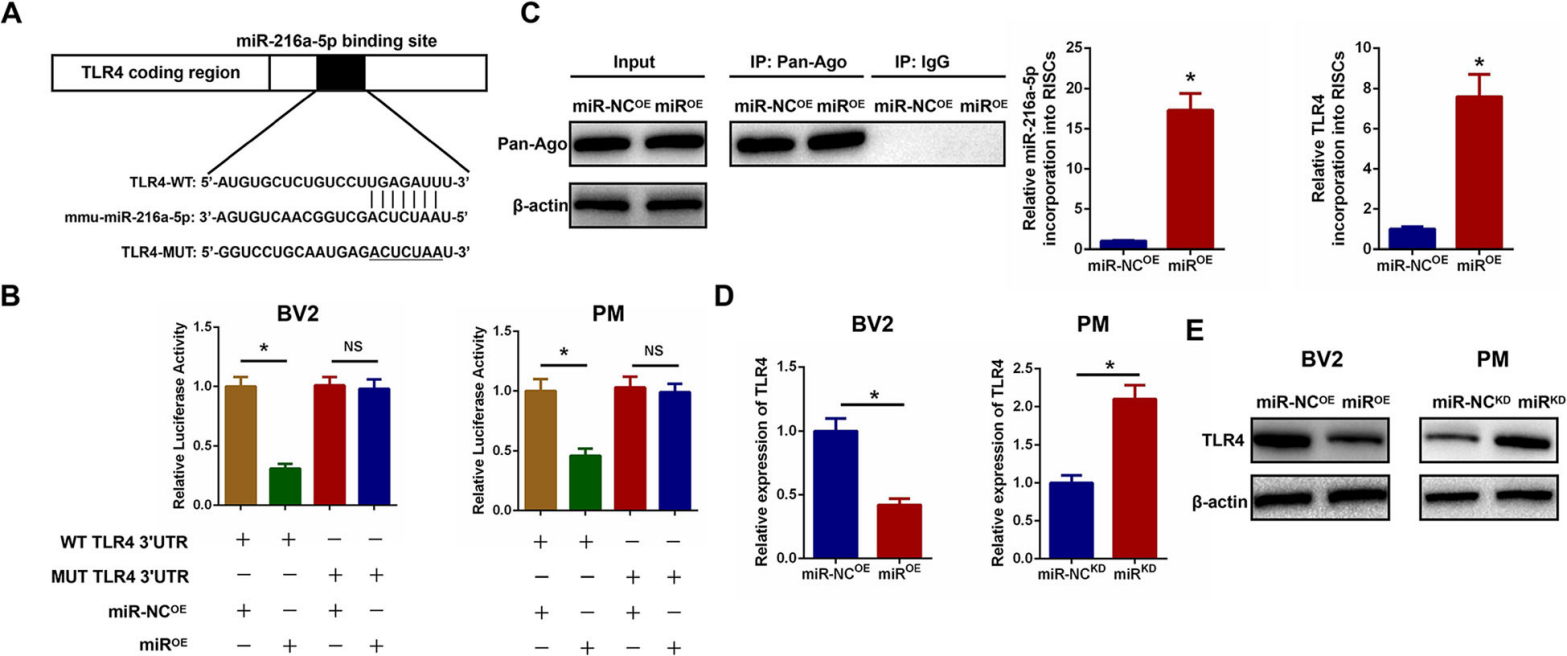
Exosomal miR-216a-5p regulates TLR4 by directly targeting the 3′-UTR. **a** and **b** Luciferase reporter assay was performed to confirm that TLR4 is the target gene of miR-216a-5p. **c** Immunoprecipitation of the Ago2/RISC (RNA-induced silencing complex) using the Pan-Ago2 antibody in BV2 microglia overexpressing miR-NC or miR-216a-5p. IgG was used as a negative control and β-actin was used as an internal control. **d** The mRNA expression level of TLR4 in microglia after miR-216a-5p overexpression and knockdown by qRT-PCR. **e** The protein expression level of TLR4 in microglia after miR-216a-5p overexpression and knockdown by western blot analysis. ^*^P < 0.05

To further investigate the potential mechanism of action of exosomal miR-216a-5p in the HExos modulation of microglial polarization, we focused on the miR-216a-5p target gene within microglia. According to the online database of miRNA targets, we found that TLR4 may be the potential target relative to inflammation attenuation (Additional file 5: Figure S5). To verify if TLR4 3′UTR is a direct target for miR-216a-5p, both wild-type (WT) and mutated (MUT) 3′-UTR sequences of TLR4 were structured based on potential binding sites (Fig. 8a). MiR-216a-5p overexpression dramatically decreased luciferase activity when the WT-3′UTR of TLR4 was co-transfected into BV2 and primary microglia compared with that in the control. No marked inhibitory effect of miR-216a-5p on luciferase activity was observed following co-transfection with the MUT 3′-UTR of TLR4 (Fig. 8b). RNA-ChIP analysis was also used to selectively detect TLR4 mRNA abundance in the Ago2/RNA-induced silencing complex (RISC) after miR-216a-5p overexpression (Fig. 8c). Enrichment in the levels of TLR4 that were incorporated into RISC was observed in miR-216a-5p-overexpressing cells. Furthermore, we observed that miR-216a-5p overexpression decreased TLR4 mRNA and protein expression levels and knockdown of miR-216a-5p increased TLR4 mRNA and protein expression levels (Fig. 8d, e), which further confirmed that TLR4 was the downstream target gene of miR-216a-5p.

### Exosomal miR-216a-5p regulates microglia M1/M2 polarization by targeting TLR4

**Fig. 9:**
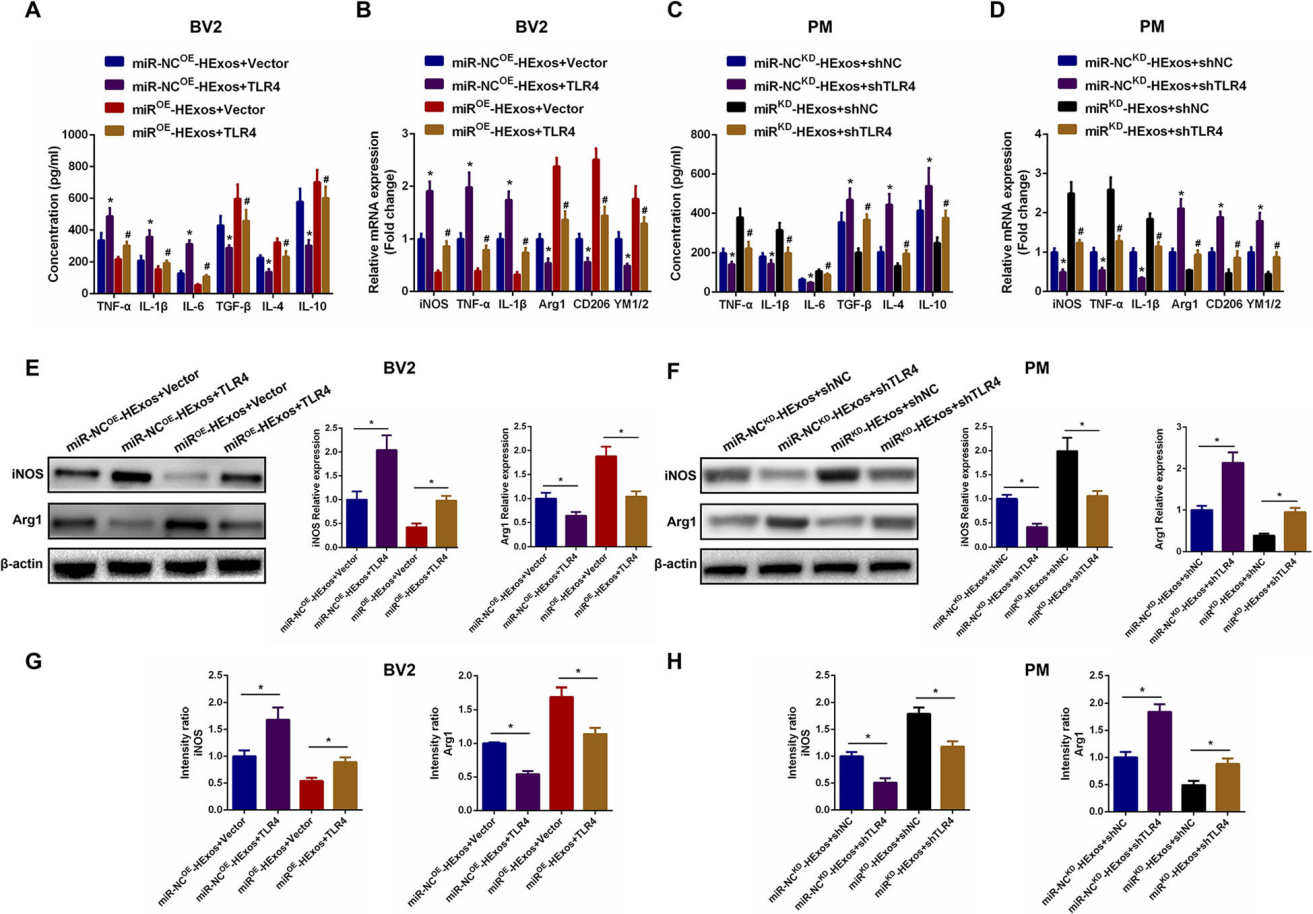
Exosomal miR-216a-5p regulates microglial M1/M2 polarization by targeting TLR4. **a**-**h** A series of gain- and loss-of-function experiments including ELISA (**a** and **c**), qRT-PCR (**b** and **d**), western blot (**e** and **f**) and immunofluorescence (**g** and **h**) were carried out to verify the functional role of TLR4 on microglial polarization in BV2 and primary microglia. **a** and **c** ELISA assays were conducted to evaluate pro- and anti-inflammatory cytokines. Rescue experiments for miR-216a-5p overexpression were carried out by the ectopic expression of TLR4 in BV2 microglia. Rescue experiments for miR-216a-5p inhibition were conducted by downregulating TLR4 in primary microglia. Microglial M1/2 polarization was detected by qRT-PCR (**b** and **d**), western blot analysis (**e** and **f**) and immunofluorescence (**g** and **h**). ^*^P < 0.05 and ^#^*P* < 0.05

To further explore the relationship between exosomal miR-216a-5p and TLR4, a series of in vitro gain- and loss-of-function experiments were conducted. To verify that the effects of exosomal miR-216a-5p on regulating microglia phenotype shift were mediated by regulation of TLR4, we overexpressed TLR4 by transfection with a TLR4 lentivirus and silenced endogenous TLR4 expression by using shRNA technology in BV2 and primary microglia. As shown in Fig. 9a, c, overexpression of TLR4 promoted the release of pro-inflammatory cytokines and inhibited the release of anti-inflammatory cytokines, while knockdown of TLR4 showed the opposite effect. Subsequently, we investigated the ability of TLR4 to counteract the effects of miR^OE^-HExos. The results demonstrated that ectopic TLR4 expression effectively promoted the release of pro-inflammatory cytokines and inhibited release of anti-inflammatory cytokines induced by administration of miR^OE^-HExos. Similarly, the unfavorable effects of administration of miR^KD^-HExos were counteracted by TLR4 downregulation in primary microglia. We continued to detect the microglial M1- and M2-related genes by qRT-PCR, western blot analysis, and immunofluorescence. Results demonstrated that TLR4 overexpression could effectively reverse the microglial phenotype induced by administration of miR^OE^-HExos and promote M2 towards M1 polarization in BV2 microglia (Fig. 9b, e, g and Additional file 6: Figure S6A). Similarly, the detrimental effects of administration of miR^KD^-HExos were counteracted by knockdown of TLR4 in primary microglia (Fig. 9d, f, h and Additional file 6: Figure S6B). These findings are consistent with our hypothesis and confirmed that exosomal miR-216a-5p regulates microglial M1/M2 polarization by targeting TLR4.

### Exosomal miR-216a-5p regulates microglia M1/M2 polarization through TLR4/NF-κB/PI3K/AKT signaling cascades

**Fig. 10:**
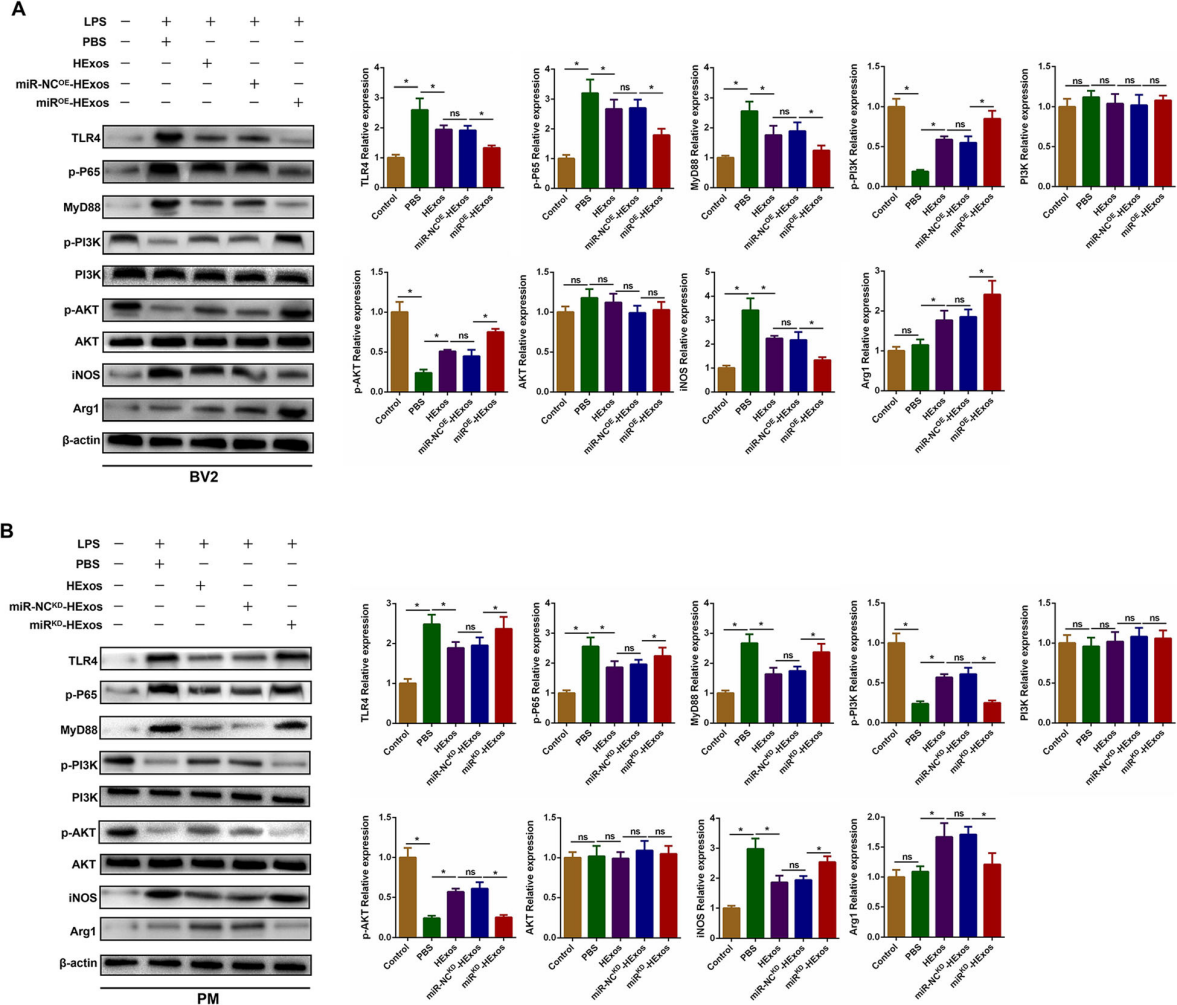
Exosomal miR-216a-5p regulates microglial M1/M2 polarization through TLR4/NF-κB/PI3K/AKT signaling cascades. **a** Representative images and quantification of western blots for TLR4 and downstream MyD88/NF-κB and PI3K/AKT signaling cascades in LPS-stimulated BV2 microglia when administrating PBS, HExos, miR-NC^OE^-HExos and miR^OE^-HExos. **b** Representative images and quantification of western blots for TLR4 and downstream MyD88/NF-κB and PI3K/AKT signaling cascades in LPS-stimulated primary microglia when administrating PBS, HExos, miR-NC^KD^-HExos and miR^KD^-HExos

Previous studies have shown that there is a mutual regulation of TLR4/NF-κB and PI3K/AKT signaling pathways in several kinds of injury/reperfusion injury [62, 63]. Inhibition of the TLR4-mediated signaling pathway may increase activation of the PI3K/AKT signaling pathway, which plays an important role in the shift to the anti-inflammatory M2 phenotype in macrophages/microglia. Therefore, we explored the possible underlying crosstalk between TLR4/NF-κB and PI3K/AKT signaling pathways after administration of HExos, miR-NC^OE^-HExos, and miR^OE^-HExos in BV2 cells. Western blot analysis revealed that the levels of TLR4, p-P65, MyD88, and iNOS were significantly downregulated and the expression levels of p-PI3K, p-AKT, and Arg1 were markedly upregulated with miR^OE^-HExos treatment (Fig. 10a). Meanwhile, with administration of miR^KD^-HExos in primary microglia, the expression levels of TLR4, p-P65, MyD88, iNOS, p-PI3K, p-AKT, and Arg1 were the opposite (Fig. 10b). These studies suggest that exosomal miR-216a-5p was involved in the HExos-mediated microglial polarization by targeting the TLR4/NF-κB/PI3K/AKT signaling cascades.

## Discussion

SCI is a traumatic intractable condition with a high rate of disability and mortality. Currently, the main focus is on the secondary period of SCI and treatments used to suppress neuroinflammation and, as a result, create a beneficial micro-environment for neurogenesis and axonal regeneration [3, 5]. To date, although great efforts have been made, the effects of SCI treatment are still limited.

Over the past decade, stem cell transplantation and therapy have shown potential for treatment of various diseases in the clinical setting, with the rapid development of regenerative medicine [18]. Stem cells have the ability to self-replicate, differentiate, and regulate hematopoietic and immune cells with great therapeutic potential. Transplantation of MSCs following SCI can create a favorable micro-environment for axonal regeneration and protect existing neurons from secondary cell death. However, several studies have shown that transplantation of MSCs after SCI increased the possibility of some side effects [64–66]. In fact, due to the existence of the BBB, few transplanted MSCs were able to reach the target injured spinal cord after intravenous administration. As a result, several challenges remain to be overcome before these MSC therapies can be applied.

Previous studies have shown that the paracrine mechanisms may account for the therapeutic effects of transplanted MSCs [23, 25]. MSC-derived exosomes have similar or better therapeutic effects in several diseases (osteonecrosis, liver/renal failure, traumatic brain/spinal cord injury, myocardial infraction, ischemic diseases, and chronic cutaneous wounds) [24, 39, 49, 67–69]. As well as a potential therapy, exosomes can potentially also overcome some of the limitations observed with direct MSCs transplantation. Since exosomes are nano-sized and membrane-permeable, studies have gained insight into the possibility of applying exosomes as nucleic acid or drug delivery carriers to cross BBB [70–73]. Zhuang et al. demonstrated that exosomes could cross the BBB obstacle and be taken up by microglia [74]. Another study suggested that exosomes loaded with anti-cancer drugs including paclitaxel and doxorubicin have potential for brain delivery across BBB in a zebrafish model [75]. Similarly, Haney et al. reported delivery of antioxidant protein catalase to the brain across BBB using exosomes in a Parkinson’s disease model [76].

Oxygen concentrations in vivo under physiological conditions differ from those in in vitro culture medium and in vitro normoxia conditions do not mimic real hypoxia micro-environments in vivo. Recent studies have shown that MSCs under hypoxia are able to enhance their biological function and increase their therapeutic effects [42, 46]. Because the hypoxic micro-environment is a prominent feature of various inflammatory and diseased tissues, including SCI, these interactions must be evaluated following both normoxic and hypoxic cell conditioning. Taken together, we established an SCI model in mice and hypothesized that exosomes derived from MSCs under hypoxic conditions could exert a better therapeutic effect compared with those under normoxic conditions. In this study, we carried out a series of experiments in vivo and in vitro to verify our hypothesis. Firstly, the results of TEM and NTA showed no morphological differences between the Exos and HExos groups with regard to their size, shape, or electron density. However, further studies revealed that hypoxic conditions could promote exosome release from MSCs and that HExos are more easily taken up by microglia. In our preliminary in vivo studies, we demonstrated that transplantation of both Exos and HExos could promote functional behavioral recovery and suppress neuroinflammation in mice following SCI and that these beneficial therapeutic effects were more evident after treatment with HExos compared with Exos.

For our in vitro experiments, in order to mimic the process of neuroinflammation in vivo, we chose two types of microglia including BV2 microglia and primary microglia and used LPS to activate microglial neuroinflammation in vitro. It is accepted that microglia can exert double-edged effects dependent on their intrinsic subtypes, including pro-inflammatory M1 phenotype and anti-inflammatory M2 phenotype [13, 77]. A previous study demonstrated that administration of IL-4 could obviously reduce tissue damage and promote functional recovery via shifting microglia to the M2 phenotype after SCI [78]. Li et al. proved that shifting microglia from M1 to M2 phenotype might be a promising therapeutic strategy to reduce neuroinflammation and improve motor recovery after brain injury [49]. Another study testified that M2 microglia could enhance primary neurons neurite length by secreting neurotropic factors [79]. Also, Yu et al. demonstrated that miR-124 could ameliorate inflammation by modulating microglial polarization in intracerebral hemorrhage [80]. Xiao et al. reported that chitinase1 might exert protective effects against Alzheimer’s disease by polarizing microglia towards the M2 phenotype [81]. Taking the role of the M2 phenotype with characteristics of anti-inflammation and neuroprotection into consideration, therapeutic treatments that can shift differentiated microglia from the M1 towards the M2 phenotype are encouraged in traumatic neuro-diseases [11, 48–50]. In this study, administration of HExos was shown to promote microglial M2 polarization and produce anti-inflammatory cytokines in vivo and in vitro. Based on these in vitro and in vivo results, we can conclude that HExos could be a promising effective bioagent to improve the functional behavioral recovery by shifting microglial M1/2 phenotype following SCI in mice.

As we have observed the beneficial effects that HExos exhibited compared with Exos, we tried to determine the underlying mechanism that contributed to the differences between Exos and HExos. Several studies have reported that exosomes derived from MSCs exert their biological functions on target cells by the delivery of specific miRNAs. A recent study demonstrated, with a myocardial infarction model, that the beneficial effects of MSC transplantation are mediated by exosomes, which shuttled miR-125b-5p by targeting Bnip3 [82]. It has also been found that BMSCs protect against I/R by secreting exosomes loaded with miR-199a-5p by targeting BIP [83]. Another study showed that BMSC-derived exosomes could modulate age-related insulin resistance by the transfer of functional exosomal miR-29b-3p and thereby inhibit the expression of the target gene SIRT1 [84]. However, an unbiased analysis of the miRNA profile of mouse HExos and a mechanistic study of the miRNA-mediated effects of shifting microglial phenotype after SCI have not been reported. Our miR-array experiments showed that miR-216a-5p was highly expressed in HExos compared with Exos and that exosomal miR-216a-5p could be transferred efficiently to the target microglia following treatment with HExos. Thus, HExos were enriched with miR-216a-5p, which could contribute to the biological differences between Exos and HExos. Through a series of in vitro and in vivo experiments, we showed that knockdown of miR-216a-5p in HExos could abolish the favorable effects of HExos in the treatment of SCI and overexpression of miR-216a-5p in HExos exerts increased favorable effects. Taken together, we can conclude that HExos enriched with miR-216a-5p could promote microglial shifting from M1 to M2 phenotype and promote neurological recovery following SCI and that HExos can act as biological vectors for the delivery of biologically functional miR-216a-5p into recipient microglia.

To better understand the underlying mechanism of exosomal miR-216a-5p, we then used bioinformatic tools to identify the potential target gene of miR-216a-5p. As a result, we chose TLR4 for further study. We verified this target gene by using luciferase report analysis and RNA-ChIP analysis. With western blot analysis, we found that the TLR4 protein level was downregulated when overexpressing miR-216a-5p and upregulated with the knockdown of miR-216a-5p in microglia, which further confirmed that TLR4 was the target downstream gene of miR-216a-5p. Toll-like receptors are the first-line molecules that initiate innate immune responses [85]. Of more than a dozen mammalian TLRs, TLR4 has been shown to be expressed in microglia and plays a vital role in CNS diseases [86, 87]. TLR4-dependent activation of microglia is crucial in degenerative and traumatic CNS diseases including Parkinson’ s disease, Alzheimer’s disease, and brain/spinal cord injury [88–90]. The TLR4 signaling pathway also plays an important role in the activation and polarization of microglia and, as a result, exerts detrimental effects on the neuronal regeneration micro-environment in CNS diseases [90, 91]. In our study, to further ensure TLR4 as the target gene of the identified miRNA, we carried out a series of gain- and loss-of-function experiments. The results demonstrated that knockdown of TLR4 in primary microglia could reverse the unfavorable effects caused by suppressing the expression of miR-216a-5p in HExos while overexpression of TLR4 in BV2 microglia could abolish the beneficial effects observed from overexpression of miR-216a-5p in HExos. Taken together, we can conclude that exosomal miR-216a-5p derived from hypoxic MSCs can suppress microglial-induced neuroinflammation by promoting microglia from M1 to M2 polarization and inhibiting the TLR4 signaling pathway in the process.

Increasing evidence has shown that there is crosstalk between the TLR4 and PI3K/AKT signal pathways [63, 92, 93]. Suppressing TLR4 signaling could activate the PI3K/AKT signaling pathway, which is indispensable for polarization of microglia/macrophages towards the M2 phenotype [62]. Thus, to better understand the relationship between these two signaling pathways after exosome addition, we used western blot analysis to detect the changes in protein level in the two signaling pathways. We found that the PI3K/AKT pathway was activated and the TLR4/NF-κB pathway inhibited following treatment with HExos and that these changes were more evident when administering miR^OE^-HExos. However, the effects of inhibiting the TLR4/NF-κB pathway and activating the PI3K/AKT pathway were partially abolished when administrating miR^KD^-HExos. These results strongly indicated that miR-216a-5p was shuttled in the HExos-mediated microglia polarization from M1 to M2 phenotype by inhibiting the TLR4/NF-κB and activating the PI3K/AKT signaling cascades. However, further experiments should be carried out to explain the underlying mechanism of the crosstalk between the TLR4/NF-κB and PI3K/AKT signaling pathways.

Although our results implied the crucial role of exosomal miR-216a-5p derived from hypoxic MSCs in regulating microglial polarization by inhibiting TLR4/NF-κB and activating the PI3K/AKT signaling pathway, we cannot rule out other possible genes that may act alone or in combination with HExos to exhibit therapeutic effects, as there were a total of 80 miRNAs that were upregulated in HExos and only the top five were selected for further study. Meanwhile, we noticed that 46 miRNAs were downregulated in HExos and these downregulated miRNAs may also contribute to these observed therapeutic effects. The precise mechanism of action of exosomes derived from hypoxic conditions in the promotion of functional behavioral recovery after SCI in mice will be explored in future studies.

In summary, our study showed that exosomes derived from hypoxic MSCs could shift microglia from M1 to M2 phenotype by transferring miR-216a-5p, which can inhibit the activation of the TLR4 pathway. As these nano-sized exosomes show promising potential as an effective intervention for the delivery of therapeutic administrations such as miRNAs and mRNAs into the injured spinal cord across BBB, the combination of miRNAs and MSC-derived exosomes may be a minimally invasive approach for the treatment of SCI.

## Conclusions

In conclusion, our study has highlighted an underlying mechanism by which cell-free exosomes derived from hypoxic MSCs promote functional behavioral recovery, by shuttling miR-216a-5p, following SCI in mice. The enriched levels of exosomal miR-216a-5p improved the therapeutic potential by shifting microglia from the M1 pro-inflammatory phenotype to the M2 anti-inflammatory phenotype by inhibiting TLR4/NF-κB and activating the PI3K/AKT signaling pathway. Hypoxic preconditioning represents a promising and effective approach to optimize the therapeutic effects of MSC-derived exosomes and a combination of miRNAs and MSC-derived exosomes may be a promising minimally invasive approach for the treatment of SCI.

## Supplementary information

**Additional file 1: Figure S1.** Identification of BMSCs (A) BMSCs exhibited a characteristic spindle-like morphology. (B) BMSCs showed potential differentiation capacity for osteogenesis, adipogenesis and chondrogenesis. (C) Flow cytometric analysis of characteristic BMSC cell surface markers (CD34, CD45, CD73 and CD105).

**Additional file 2: Figure S2.** HExos promoted the expression of Arg1 and inhibited the expression of iNOS. (A) Immunofluorescence staining of Iba1, iNOS and Arg1 in Control, PBS, Exos and HExos groups in BV2 microglia. (B) Immunofluorescence staining of Iba1, iNOS and Arg1 in Control, PBS, Exos and HExos groups in primary microglia.

**Additional file 3: Figure S3.** Exosomal miR-216-5p promoted functional behavioral recovery and reduced lesion area following SCI in vivo. (A) BMS was used to functionally grade the mice in the miR-NC^OE^-HExos, miR^OE^-HExos, miR-NC^KD^-HExos and miR^KD^-HExos groups up to 28 days post-injury (*n* = 8/group). (B-D) Representative footprints of an animal walking 28 days after SCI and quantification of the footprints analysis findings in each mouse. Blue: frontpaw print; red: hindpaw print (n = 8/group). (E) Representative immunostaining images of GFAP of spinal cord at day 28 post-injury (n = 8/group). (F) Quantification of lesion volumes in miR-NC^OE^-HExos, miR^OE^-HExos, miR-NC^KD^-HExos and miR^KD^-HExos groups.

**Additional file 4: Figure S4.** Exosomal miR-216a-5p promoted the expression of Arg1 and inhibited the expression of iNOS. (A) Immunofluorescence staining of Iba1, iNOS and Arg1 in miR-NC^OE^-HExos and miR^OE^-HExos groups in BV2 microglia. (B) Immunofluorescence staining of Iba1, iNOS and Arg1 in miR-NC^KD^-HExos and miR^KD^-HExos groups in primary microglia.

**Additional file 5: Figure S5.** Overview of bioinformatics analysis showing TLR4 as a downstream target of miR-216a-5p.

**Additional file 6: Figure S6.** Exosomal miR-216a-5p promoted the expression of Arg1 and inhibited the expression of iNOS by inhibiting TLR4. (A) Immunofluorescence staining of Iba1, iNOS and Arg1 in miR-NC^OE^-HExos+Vector, miR-NC^OE^-HExos+TLR4, and miR^OE^-HExos+Vector and miR^OE^-HExos+TLR4 groups in BV2 microglia. (B) Immunofluorescence staining of Iba1, iNOS and Arg1 in miR-NC^KD^-HExos+shNC, miR-NC^KD^-HExos+shTLR4, miR^KD^-HExos+shNC and miR^KD^-HExos+ shTLR4 groups in primary microglia.

**Additional file 7: Table S1.** The primer sequences used in this work.

## Data Availability

Most of the datasets supporting the conclusions of this article are included within this article and the additional files. The datasets used or analyzed during the current study are available on reasonable request.

## Abbreviations

BBB: Blood-brain barrier
CNS: Central nervous system
Exos: Exosomes derived from MSCs under normoxia
HExos: Exosomes derived from MSCs under hypoxia
MEP: Motor-evoked potential
miRNA: MicroRNA
MSCs: Mesenchymal stem cells
PM: Primary microglia
SCI: Spinal cord injury
TLR4: Toll-like receptor 4
