# Supplementary figures and images for: Exosome-shuttled miR-216a-5p from hypoxic preconditioned mesenchymal stem cells repair traumatic spinal cord injury by shifting microglial M1/M2 polarization
Source: J Neuroinflammation. 2020 Feb 4;17:47. doi: 10.1186/s12974-020-1726-7 (PMC7001326; doi:10.1186/s12974-020-1726-7)

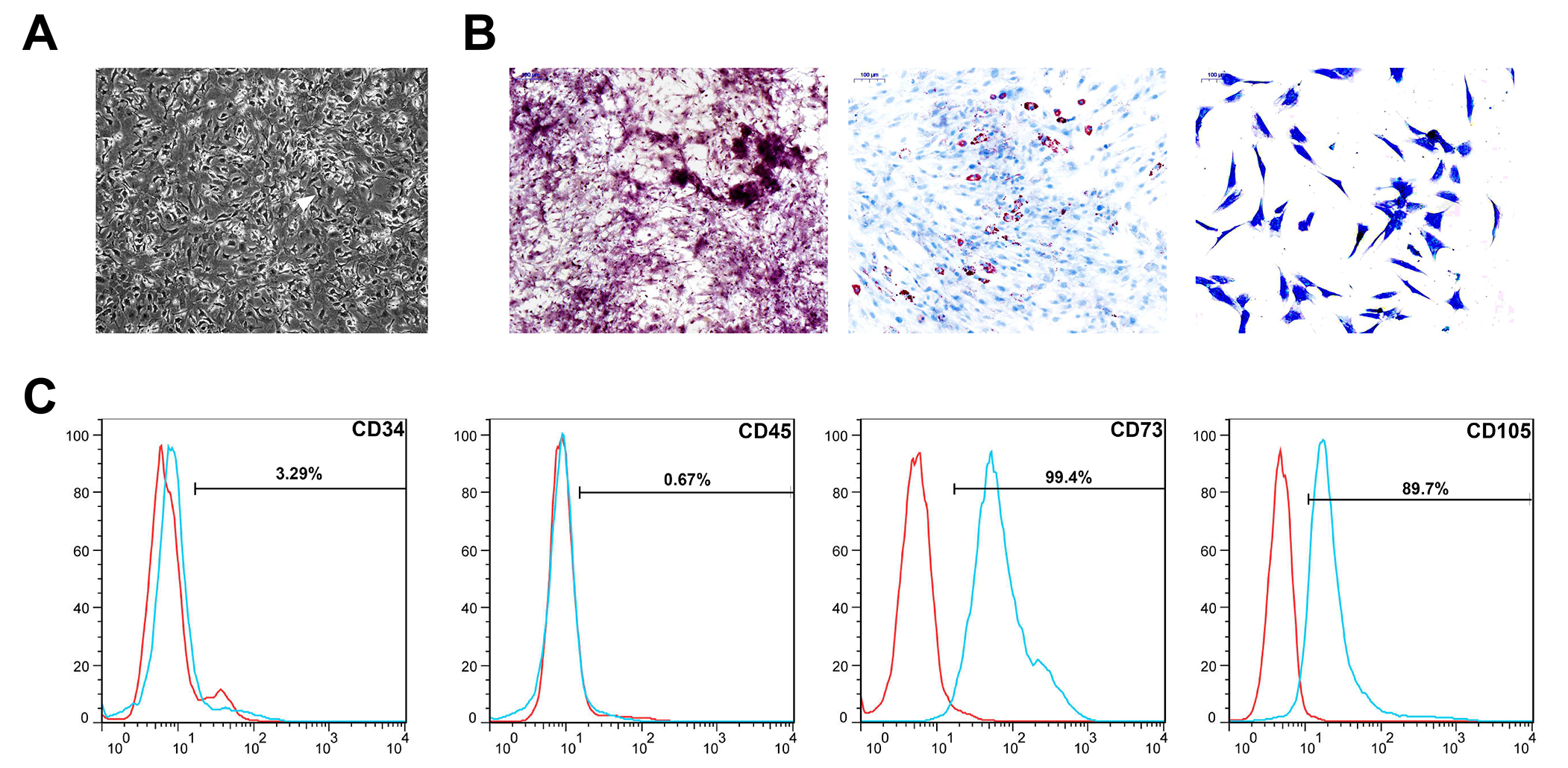

Supplement: Supplementary file 1 — Additional file 1: Figure S1. Identification of BMSCs (A) BMSCs exhibited a characteristic spindle-like morphology. (B) BMSCs showed potential differentiation capacity for osteogenesis, adipogenesis and chondrogenesis. (C) Flow cytometric analysis of characteristic BMSC cell surface markers (CD34, CD45, CD73 and CD105). [file 12974_2020_1726_MOESM1_ESM.tif]

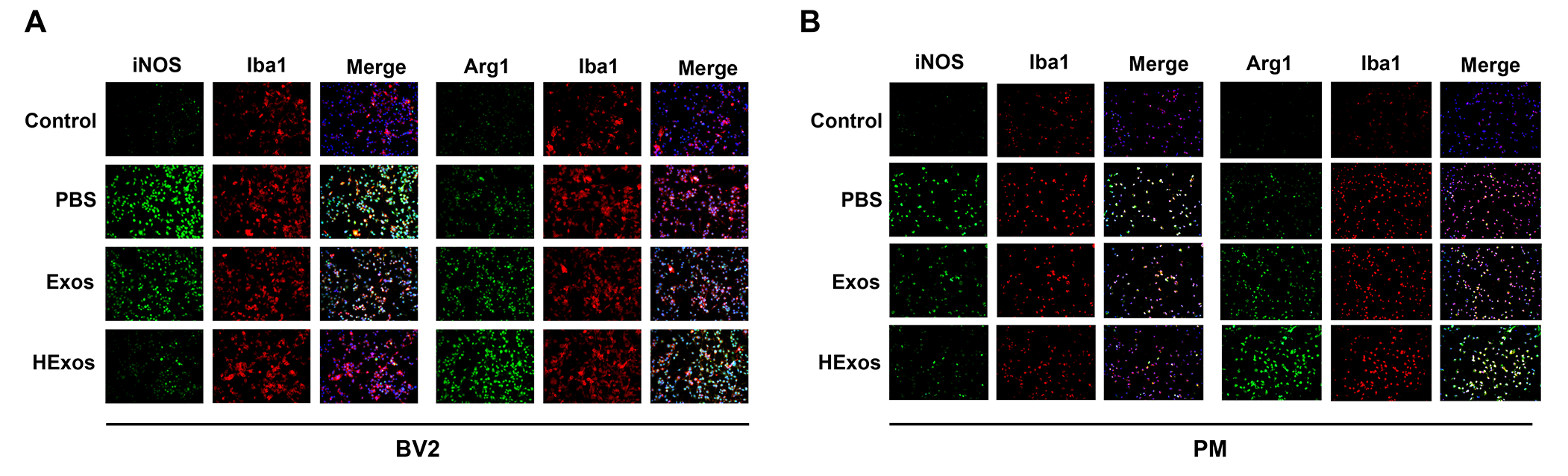

Supplement: Supplementary file 2 — Additional file 2: Figure S2. HExos promoted the expression of Arg1 and inhibited the expression of iNOS. (A) Immunofluorescence staining of Iba1, iNOS and Arg1 in Control, PBS, Exos and HExos groups in BV2 microglia. (B) Immunofluorescence staining of Iba1, iNOS and Arg1 in Control, PBS, Exos and HExos groups in primary microglia. [file 12974_2020_1726_MOESM2_ESM.tif]

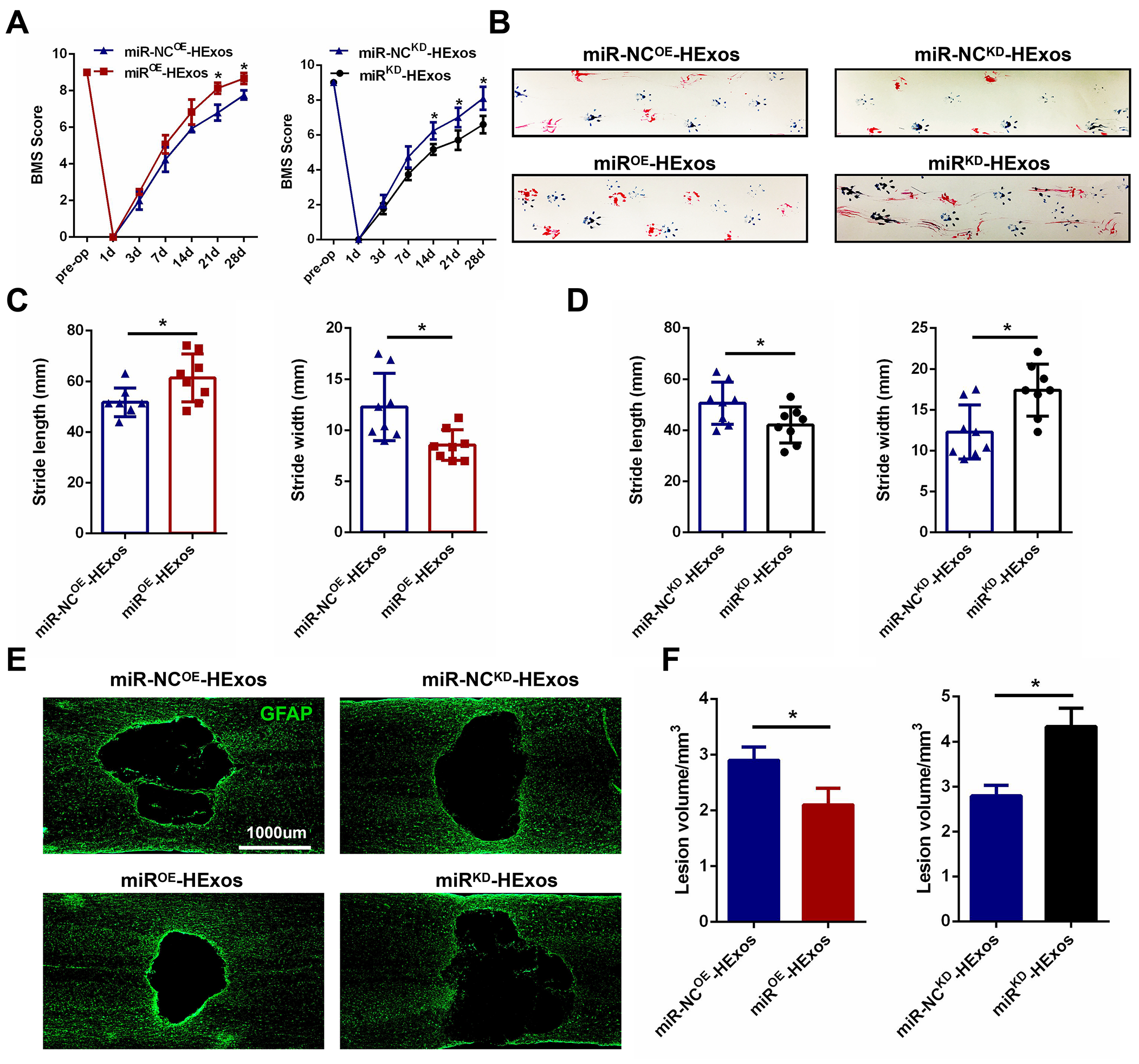

Supplement: Supplementary file 3 — Additional file 3: Figure S3. Exosomal miR-216-5p promoted functional behavioral recovery and reduced lesion area following SCI in vivo. (A) BMS was used to functionally grade the mice in the miR-NCOE-HExos, miROE-HExos, miR-NCKD-HExos and miRKD-HExos groups up to 28 days post-injury (n = 8/group). (B-D) Representative footprints of an animal walking 28 days after SCI and quantification of the footprints analysis findings in each mouse. Blue: frontpaw print; red: hindpaw print (n = 8/group). (E) Representative immunostaining images of GFAP of spinal cord at day 28 post-injury (n = 8/group). (F) Quantification of lesion volumes in miR-NCOE-HExos, miROE-HExos, miR-NCKD-HExos and miRKD-HExos groups. [file 12974_2020_1726_MOESM3_ESM.tif]

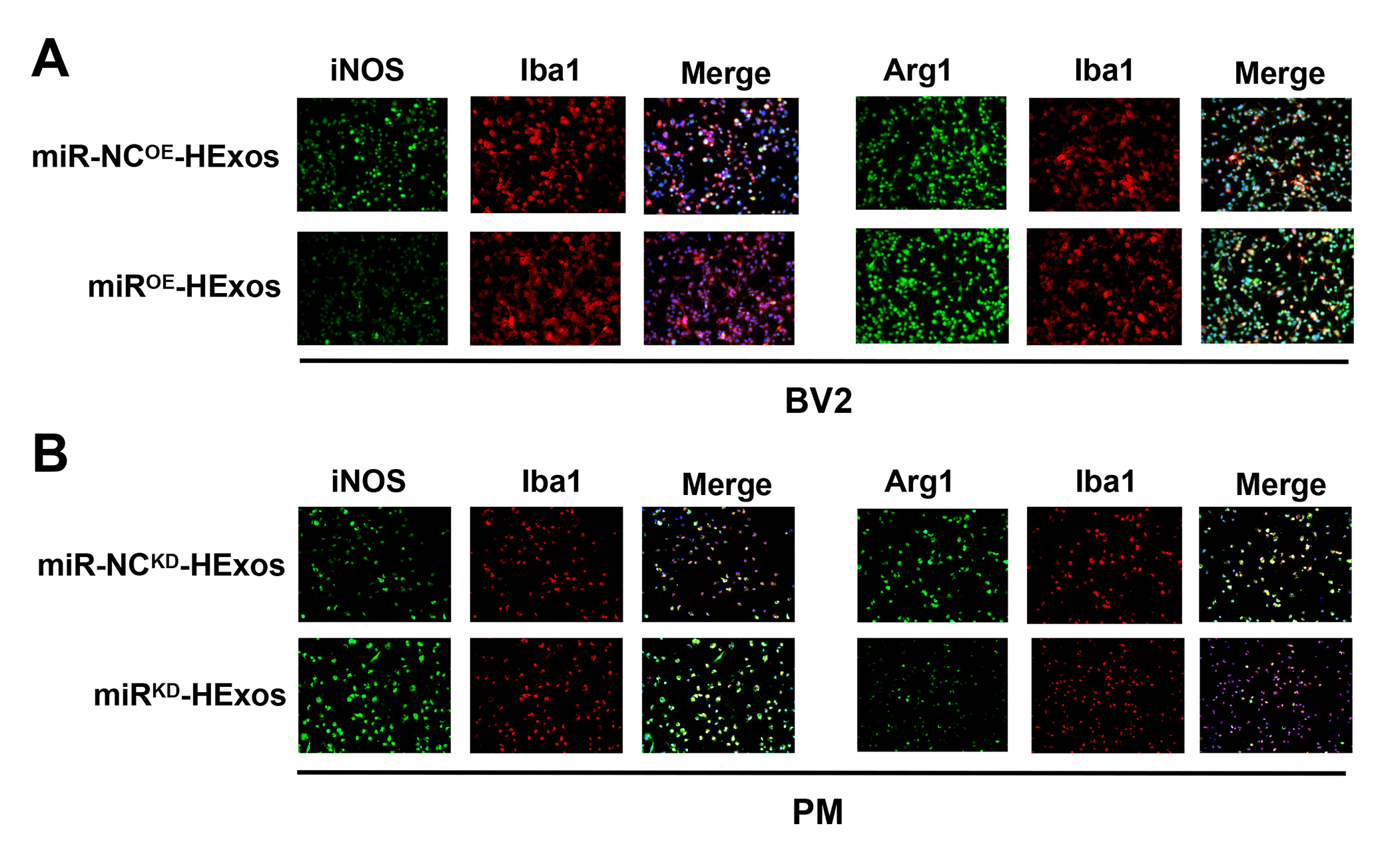

Supplement: Supplementary file 4 — Additional file 4: Figure S4. Exosomal miR-216a-5p promoted the expression of Arg1 and inhibited the expression of iNOS. (A) Immunofluorescence staining of Iba1, iNOS and Arg1 in miR-NCOE-HExos and miROE-HExos groups in BV2 microglia. (B) Immunofluorescence staining of Iba1, iNOS and Arg1 in miR-NCKD-HExos and miRKD-HExos groups in primary microglia. [file 12974_2020_1726_MOESM4_ESM.tif]

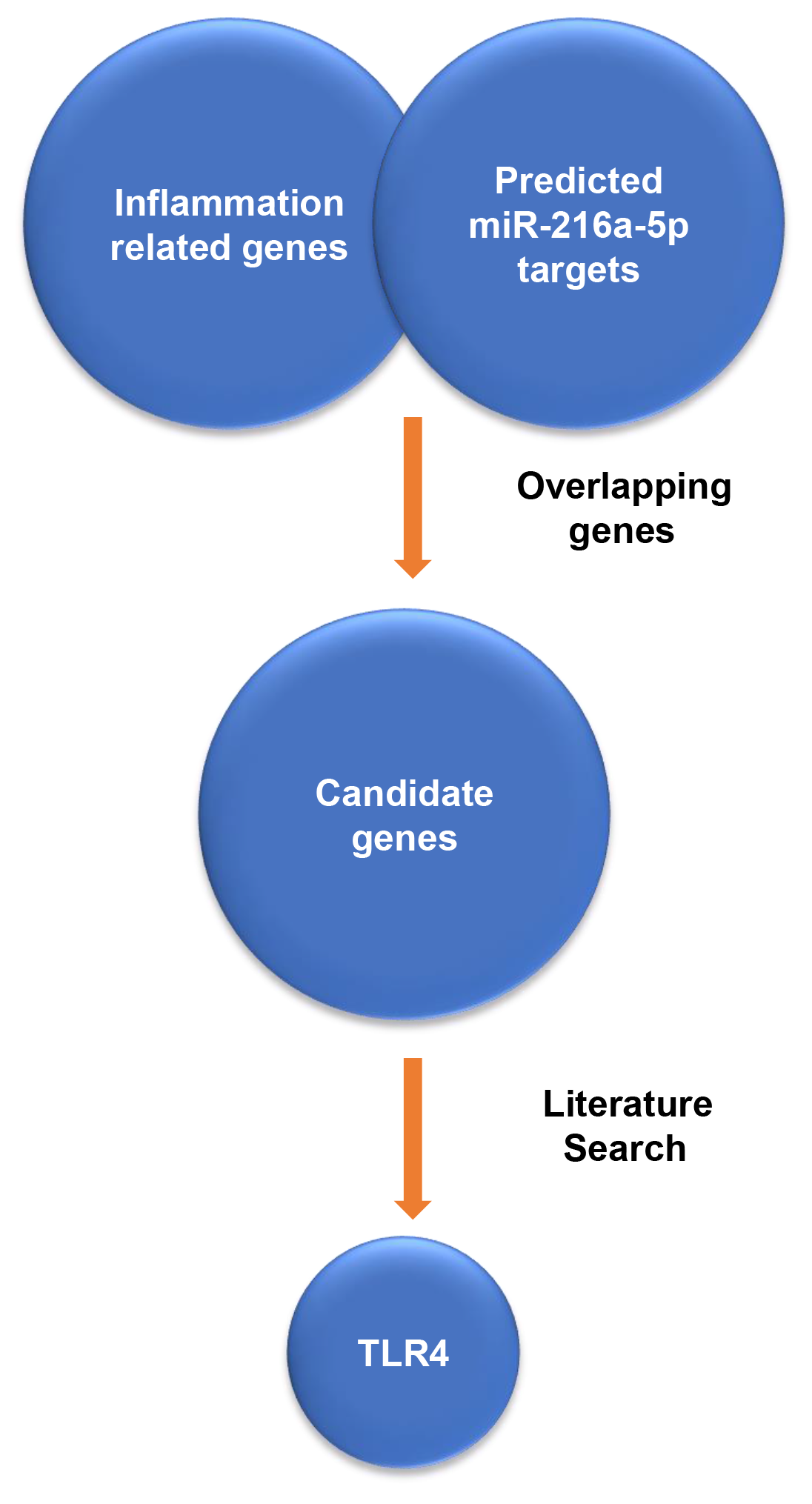

Supplement: Supplementary file 5 — Additional file 5: Figure S5. Overview of bioinformatics analysis showing TLR4 as a downstream target of miR-216a-5p. [file 12974_2020_1726_MOESM5_ESM.tif]

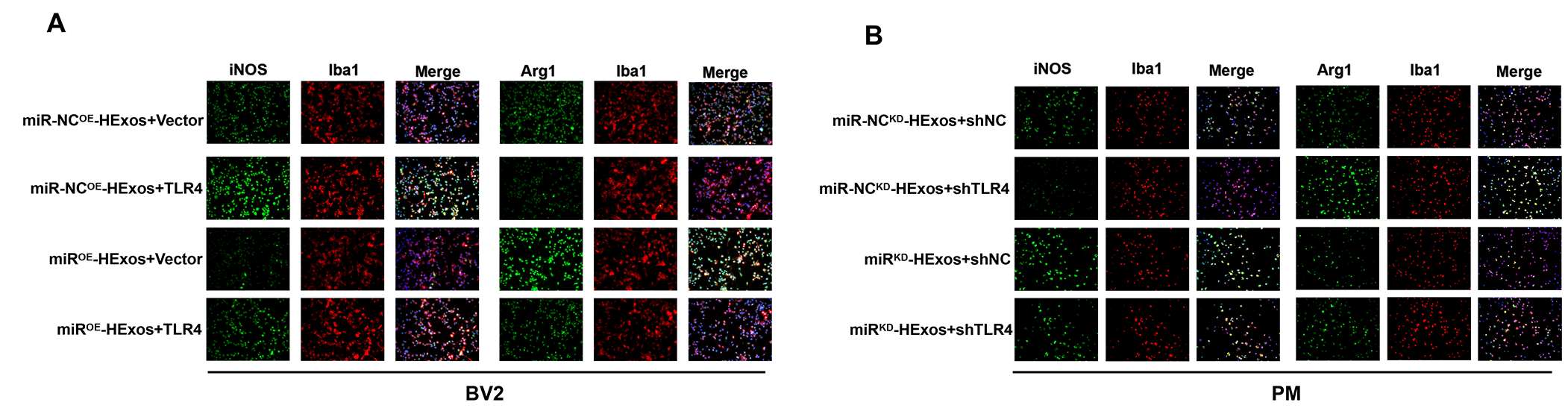

Supplement: Supplementary file 6 — Additional file 6: Figure S6. Exosomal miR-216a-5p promoted the expression of Arg1 and inhibited the expression of iNOS by inhibiting TLR4. (A) Immunofluorescence staining of Iba1, iNOS and Arg1 in miR-NCOE-HExos+Vector, miR-NCOE-HExos+TLR4, and miROE-HExos+Vector and miROE-HExos+TLR4 groups in BV2 microglia. (B) Immunofluorescence staining of Iba1, iNOS and Arg1 in miR-NCKD-HExos+shNC, miR-NCKD-HExos+shTLR4, miRKD-HExos+shNC and miRKD-HExos+ shTLR4 groups in primary microglia. [file 12974_2020_1726_MOESM6_ESM.tif]
